# Foam Replica Method in the Manufacturing of Bioactive Glass Scaffolds: Out-of-Date Technology or Still Underexploited Potential?

**DOI:** 10.3390/ma14112795

**Published:** 2021-05-24

**Authors:** Elisa Fiume, Sara Ciavattini, Enrica Verné, Francesco Baino

**Affiliations:** 1 Department of Applied Science and Technology (DISAT), Institute of Materials Physics and Engineering, Politecnico di Torino, Corso Duca degli Abruzzi 24, 10129 Torino, Italy; elisa.fiume@polito.it (E.F.); sara.ciavattini@icloud.com (S.C.); enrica.verne@polito.it (E.V.); 2 Department of Mechanical and Aerospace Engineering (DIMEAS), Politecnico di Torino, Corso Duca degli Abruzzi 24, 10129 Torino, Italy

**Keywords:** foam replica method, bioactive glasses, scaffolds, bone tissue engineering, sacrificial templates, porosity, mechanical properties, permeability, in vivo studies

## Abstract

Since 2006, the foam replica method has been commonly recognized as a valuable technology for the production of highly porous bioactive glass scaffolds showing three-dimensional, open-cell structures closely mimicking that of natural trabecular bone. Despite this, there are important drawbacks making the usage of foam-replicated glass scaffolds a difficult achievement in clinical practice; among these, certainly the high operator-dependency of the overall manufacturing process is one of the most crucial, limiting the scalability to industrial production and, thus, the spread of foam-replicated synthetic bone substitutes for effective use in routine management of bone defect. The present review opens a window on the versatile world of the foam replica technique, focusing the dissertation on scaffold properties analyzed in relation to various processing parameters, in order to better understand which are the real issues behind the bottleneck that still puts this technology on the Olympus of the most used techniques in laboratory practice, without moving, unfortunately, to a more concrete application. Specifically, scaffold morphology, mechanical and mass transport properties will be reviewed in detail, considering the various templates proposed till now by several research groups all over the world. In the end, a comprehensive overview of in vivo studies on bioactive glass foams will be provided, in order to put an emphasis on scaffold performances in a complex three-dimensional environment.

## 1. Introduction to the Foam Replica Method

The foam replica method was developed by Schwartzwalder and Somers in 1963 for the manufacturing of ceramic foams [1]. 

Chen et al. [2] and Legeros et al. [3] were the first, in 2006, to introduce this technique in bone tissue engineering (BTE) for the development of bioactive glass (BG) scaffolds. Since that moment, the foam replica method has been considerably used by several research groups to obtain porous glass scaffolds for bone regenerative applications. 

The technique is based on the replication of the porous structure of a sacrificial template in order to obtain its positive replica made of glass or glass–ceramic particles, which are later sintered around the organic strut.

In a typical process, the foam is dipped in a slurry consisting of glass powders suspended into a binder solution to obtain a consistent coating on the foam struts [4,5]. 

The characteristics of the final object, such as mechanical properties, permeability, and porosity, can be easily adjusted according to the selected template, which defines the porous architecture of the scaffold and the mass transport phenomena [6] as well as the process parameters that can be tuned to optimize the structural properties of the final product. As an example, the coating thickness can be modulated depending on the number of consecutive immersions performed in order to improve mechanical properties. Sometimes, the sintering process can be optimized either by increasing the temperature of the treatment or by increasing the solid load of the slurry, producing an increase in the contact points between adjacent particles and, thus, a reduction in the number of inter-particle voids [7]. 

The excess slurry is removed by squeezing the foams, which are then left to dry in order to obtain the so-called green bodies. After drying, the foam is burned out by high temperature thermal treatments, typically between 300 and 600 °C, in order to minimize structural damage of the porous glass coating. Once the foam is removed, the glass struts are densified by sintering at 600–1000 °C, depending on the composition and particle size of the glass [5,8]. Frequently, the foam burning out and the glass sintering are combined in a single treatment. It is carried out by maintaining a very low heating rate uphill in order to burn out the foam without damaging the glass coating. Then the treatment involves keeping the sample at the chosen sintering temperature for a few hours in order to sinter the glass struts [9,10]. 

**Figure 1:**
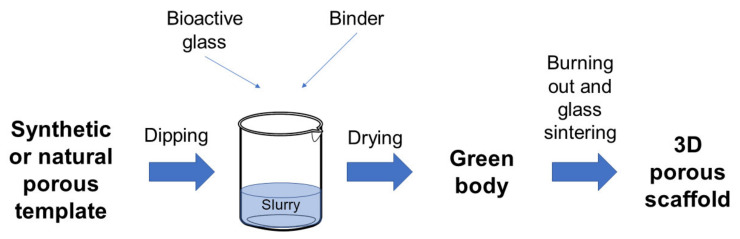
Schematic representation of the foam replica technique.

Figure 1 provides a schematization of the process.

Currently, the foam replica method is one of the most popular, affordable, relatively easy, and effective techniques for the development of highly porous and interconnected 3D scaffolds [11]. In fact, no specific equipment is required to implement the method and the only cost is related to the production and processing of the glasses used as basic material, which must be covered anyway, regardless of the manufacturing process, and the purchase of the sacrificial template, which is minimal. In addition, when variants using waste materials and natural templates are implemented, the cost of the foam replication technique can be considered almost zero.

The success of this manufacturing technique is mainly due to its versatility, which relies upon the possibility of using a wide variety of sacrificial templates of both synthetic (e.g., commercial polymeric sponges [2]) and natural (e.g., marine sponges [12]) origin, as well as the opportunity to process both traditional melt-derived and sol–gel glasses and glass–ceramics.

As an example, the foam replica method was used in combination with evaporation-induced self-assembly (EISA) [13] to fabricate hierarchical porous BG (HPBG) scaffolds, using polyurethane foam and a surfactant as co-templates for scaffold macropores and mesopores, respectively [14,15]. 

Over the last decade, natural biological materials as macroporous templates became widely used due to their easy availability and low cost, thus leading to the possibility of obtaining diverse and environment-friendly structures [16].

Despite all these appealing aspects, the foam replica method is currently affected by one of the most critical drawbacks in biomedical applications, which is a relative lack of results reproducibility and control of the macroporous architecture, partially ensured only for small and geometrically simple samples. In fact, by increasing the geometrical dimension/complexity of the specimens, as well as the number of each single batch, undesired and often uncontrollable phenomena may occur [8], such as the formation of slurry gradients associated with pore occlusion in a confined portion of volume, yielding to variable porosity between different samples. Moreover, the strong dependence of the final outcome on the operator precludes the standardization of the manufacturing process, preventing a move—unfortunately—to a more concrete application.

In addition, different from advanced additive manufacturing technologies, it is not possible to achieve pore architecture and porosity tailored to the specific patient, and the final architecture of the graft has to be optimized by a preliminary and accurate selection of the basic material, the template’s features, and the sintering conditions [17].

However, concerning the possibility of faithfully reproducing the complex 3D trabecular architecture typical of spongy bone, the foam replica method probably remains the most promising one, thus motivating further efforts toward the optimization of the process. 

## 2. Variants of the Process and Impact on the Morphological Properties

Morphological properties refer to the 3D architecture of scaffolds, in particular, to the shape, size, and interconnectivity of pores [18]. 

These properties are mainly influenced by the structure of the sacrificial template and process parameters. Some process parameters, such as sintering temperature, are in turn intimately linked to glass composition, which determines the thermal response of the glass and thus the devitrification kinetics and the scaffold densification upon thermal treatments [19]. 

In the following section, morphological properties of scaffolds produced from different sacrificial templates will be discussed in detail, paying particular attention to the macroporous, open-cell architecture. 

### 2.1. Replica of Synthetic Templates

Both Chen et al. [2] and Legeros et al. [3] developed bioactive glass scaffolds for BTE using polyurethane (PU) sponges as sacrificial templates. Since then, PU sponges have become the most widely used sacrificial templates for the production of glass-based bone-like structures. Indeed, their morphological similarity to trabecular bone in terms of open-cell architectures and high interconnectivity between adjacent pores makes them very appealing templates for the manufacturing of synthetic bone grafts; in addition, they are low cost (0.01 USD/cm^3^ [20]) and easily available on the market as air filters with different porosity levels [21,22,23]. 

This section provides an overview of the bioactive glass scaffolds produced by the foam replica method using the polyurethane sponges as sacrificial templates.

**Figure 2:**
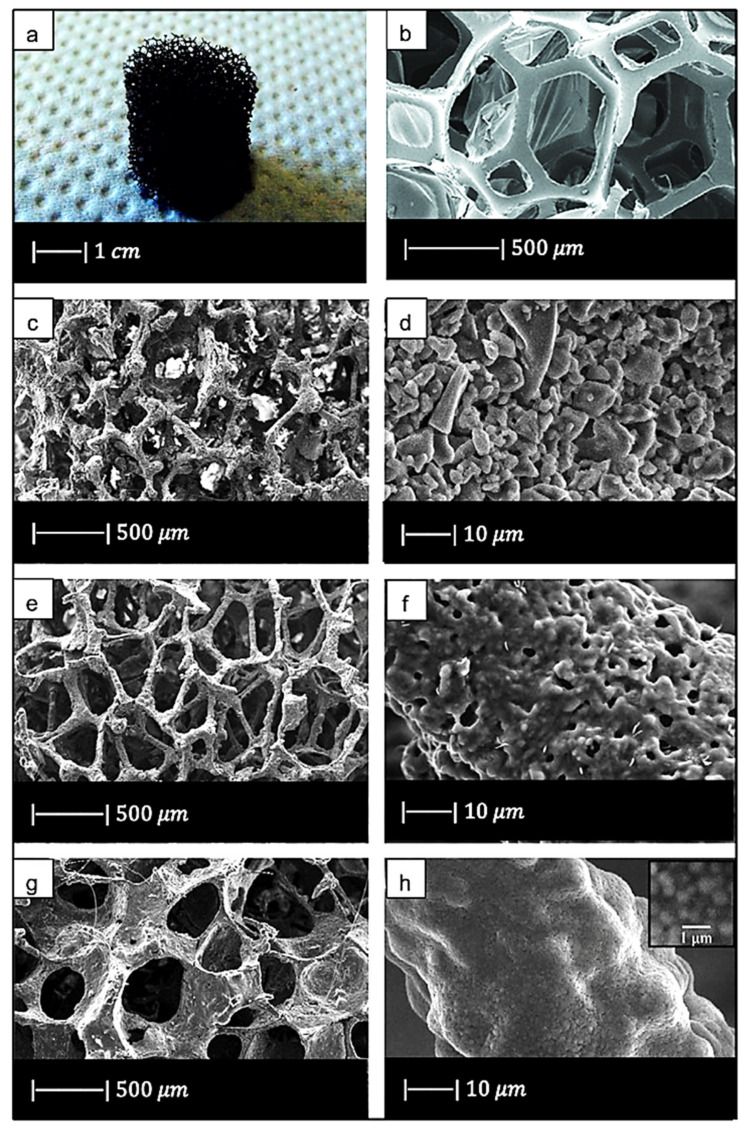
(**a**) Digital camera image of polyurethane (PU) foam and (**b**) SEM image of the PU foam reproduced respectively from Boccardi et al. [12] and Zhang et al. [24] under the terms of the Creative Commons Attribution License. (**c**–**f**) Pore structure and strut morphology of 45S5 Bioglass^®^-derived foams sintered at (**c**,**d**) 900 °C for 5 h; (**e**,**f**) 950 °C for 2 h; and (**g**,**h**) 1000 °C for 1 h. Figures from (**c**) to (**h**) reproduced with permission from Chen et al. [2] (Copyright © 2021 Elsevier Ltd.).

Chen et al. [2] developed 45S5 Bioglass^®^-based scaffolds by using totally reticulated polyester-based polyurethane foams of 60 pores per inch (ppi) from Recticel UK (Corby) as sacrificial templates (Figure 2a,b) [2].

The slurry was obtained by adding melt-derived 45S5 Bioglass^®^ powders (particle size ~5 µm) to polyvinyl alcohol (PVA)-based binder solution [2].

Three different sintering conditions were used, i.e., 900 °C/5 h; 950 °C/0–5 h; and 1000 °C/0–2 h, to investigate the effect of the thermal treatment on the porous microstructure and mechanical properties. The heating and cooling rates were 2 and 5 °C/min, respectively [2].

The macroporous network and microstructure of the scaffolds obtained under different sintering conditions are shown in Figure 2c–h.

For all the samples produced, the total porosity was ~90% and macropores were in the range of 510–720 μm, regardless of the sintering conditions.

Comparing Figure 2c,e,g, it can be observed that cell struts were much thicker when sintered at 1000 °C for up to 1 h than at 900–950 °C for 2–5 h because reaching a higher temperature allowed for greater densification of the glass.

Images at higher magnification show that, in the scaffold thermally treated at 900 °C (Figure 2d), a low sintering was achieved. Indeed, it was still possible to observe the presence of glass particles, while the scaffolds thermally treated at 950 °C (Figure 2f) exhibited greater sintering and ones treated at 1000 °C (Figure 2h) were fully sintered. In the latter, glass particles were no longer distinguishable, and the scaffold surface appeared smooth and round. Indeed, as the temperature increased, the viscosity decreased and sintering by viscous flow was favored [2].

In scaffolds sintered at 1000 °C (Figure 2h), fine crystalline grains of 0.5 μm in diameter were observed.

XRD analysis revealed that crystallization occurred in all the scaffolds [2]. This result confirmed the occurrence of a broad crystallization before a significant viscous flow sintering in 45S5 Bioglass^®^ and other bioactive glasses [2]. Bioglass^®^, in fact, crystallizes before a significant sintering level is achieved, due to the presence of a highly disrupted silica network [25].

**Figure 3:**
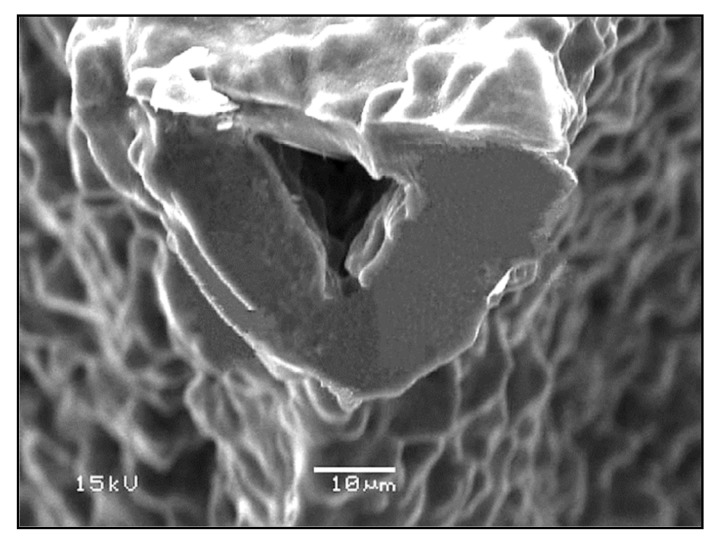
The hollow center of a single strut in a Bioglass^®^-derived foam sintered at 1000 °C for 1 h. Figure reproduced with permission from Chen et al. [2] (Copyright © 2021 Elsevier Ltd.).

In addition, due to the limited viscous flow or diffusion in 45S5 Bioglass^®^, voids resulting from the burn-out of the PU foam were not filled and remained as triangular-shaped pores in the struts (Figure 3) [4]. Analogous morphologies were obtained for several ceramic foams fabricated by the polymer sponge method [26].

Subsequently, Baino et al. [27] succeeded in obtaining well-densified Bioglass^®^ scaffolds without template-derived voids. This achievement was reached by sintering Bioglass^®^ at the temperature at which the powders began to melt. Despite the high sintering temperature used, the scaffolds were characterized by a porosity of 70%, which is sufficient for BTE applications, and a pore size of between 100 and 600 μm [27].

In the same study Baino et al. [27] used CEL2 glass to fabricate bioactive glass–ceramic scaffolds. CEL2 is a silicate glass with composition 45SiO_2_–3P_2_O_5_–26CaO–7MgO–15Na_2_O–4K_2_O mol % originally developed and investigated by Vitale-Brovarone et al. [28] at Politecnico di Torino.

The composition of the system has a strong influence on the thermal behavior of the material. Indeed, in CEL2 glass, the viscous flow sintering starts at the glass transition temperature and is totally completed before crystallization onset. For this reason, CEL2 glass allows the obtaining of scaffolds with a higher degree of sintering compared to Bioglass^®^ scaffolds [27].

In addition, these scaffolds exhibited well-interconnected pores of 100–500 μm in size [27].

CEL2 scaffolds with similar morphology were obtained by Vitale-Brovarone [29]. In this study it was found that particle size represents an important factor in obtaining an open and interconnected porosity. Indeed, CEL2 particles with a 30 μm diameter led to a lot of closed porosity in the final scaffold, while ones sieved below 30 μm gave the best sponge impregnation [29].

**Figure 4:**
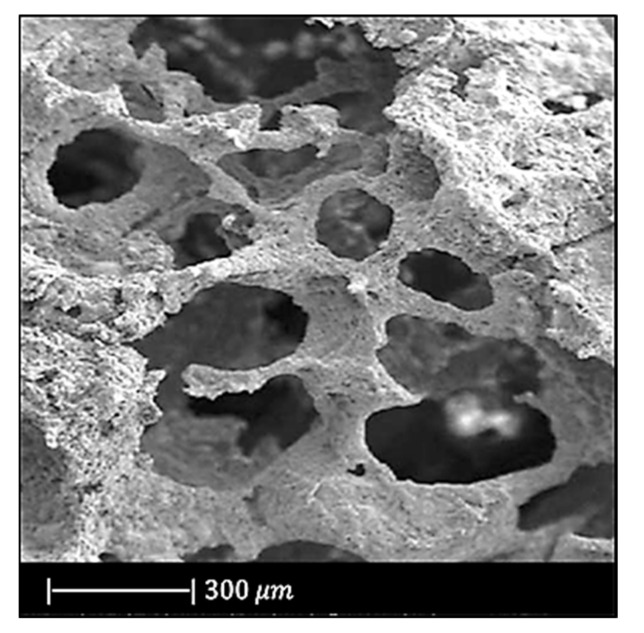
SEM image of ICEL2 scaffold. Figure adapted with permission from Baino et al. [9] (Copyright © 2021, Springer Science Business Media, LLC).

By reversing the molar quantities of SiO_2_ and P_2_O_5_ in CEL2, phosphate glass ICEL2 glass was obtained [9]. This glass is characterized by a narrower sintering window than CEL2 glass, and consequently a lower degree of sintering was reached in ICEL2 scaffolds (Figure 4) developed by Baino et al. [9] with respect to CEL2 scaffolds.

However, ICEL2 structures exhibited an effective densification of the pore struts [9].

As far as pore size is concerned, there was no difference between CEL2 and ICEL2 scaffolds [9].

The first calcium phosphate glass scaffold was developed by Legeros et al. [3]. They used a glass in the CaO–CaF_2_–P_2_O_5_–MgO–ZnO system and a reticulated polyurethane ester sponge as sacrificial template. This sponge had 500 three-dimensionally interconnected open pores per each linear mm [3].

The slurry was composed of calcium phosphate glass powders, distilled water and organic additives such as binder, dispersant, and a drying chemical control additive (DCCA). Polyvinyl alcohol (PVA), polyethylene glycol (PEG) and dimethyl formamide were chosen as binder, dispersant and drying chemical control additive, respectively [3].

Before the dipping process, the surface layer of the sponge was treated in a 2% NaOH solution ultrasonically to increase its hydrophilicity [3].

Finally, the glass was sintered for 2 h at different temperatures between 650 and 850 °C [3].

This procedure was repeated twice to thicken the framework of the scaffolds and as a result to obtain an increased compressive strength [3].

**Figure 5:**
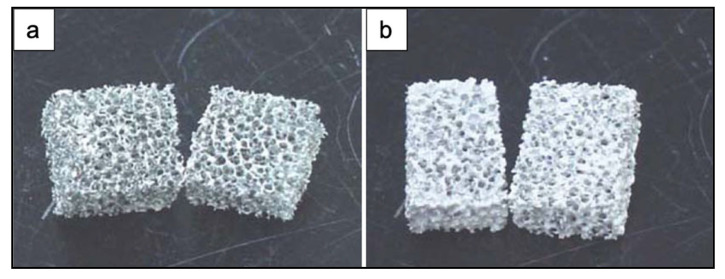
Photographs of PU ester sponge foams after sintering at 850 °C (**a**) once and (**b**) twice. Figure adapted from Park et al. [3] with permission (Copyright © 2021, Springer Science Business Media, Inc.).

Figure 5 shows the obtained scaffolds after sintering at 850 °C (a) once and (b) twice.

**Figure 6:**
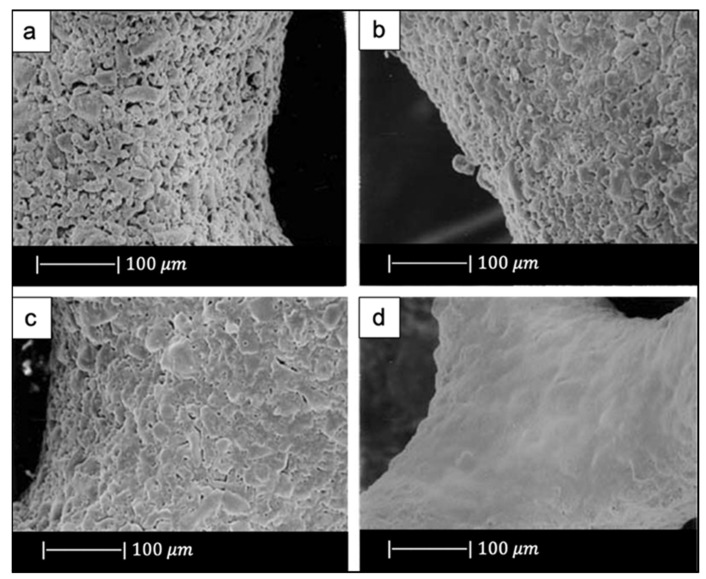
SEM images of the scaffolds sintered at (**a**) 650 °C, (**b**) 700 °C, (**c**) 800 °C, (**d**) 850 °C. Figure adapted from Park et al. [3] with permission (Copyright © 2021, Springer Science Business Media, Inc.).

The scaffolds obtained under different sintering conditions are shown in Figure 6.

The scaffolds thermally treated at 650 °C showed gray color while the ones sintered at 850 °C were white. The gray color was due to sponge remains and additives still present in the sample due to the low sintering temperature used [3].

The glass powders sintered at 650 °C had just started to bond with each other (Figure 6a). As the sintering temperature increased, the voids between the powders were reduced in number and size (Figure 6b,c). Scaffolds sintered at 850 °C showed a dense microstructure without voids and cracks (Figure 6d) [3].

The heating rate proved to be one of the most important factors in obtaining successful scaffolds. Indeed, the use of a heating rate above 3 °C/min resulted in very weak or locally collapsed scaffolds. This was probably due to the removal of the sponge before the formation of necks between particles and to an insufficient binding strength of the particles. By using a heating rate lower than 1 °C/min, a more resistant 3D structure with interconnected open pores was obtained [3]. The macropores in these scaffolds were about 500~800 μm [3].

Later, Lee et al. [30] fabricated calcium phosphate glass scaffolds having the same morphological properties as those developed by Legeros et al. [3]. This similarity is explained by the fact that both the scaffolds were made from the same glass, were sintered at the same temperature, and underwent a double coating and double sintering process.

In another study, Baino and Vitale-Brovarone [31], using an experimental silicate glass with composition 57SiO_2_–34CaO–6Na_2_O–3Al_2_O_3_ (mol %) named SCNA, obtained scaffolds with a bimodal pore size distribution: pores > 100 μm and pores < 10–20 μm, which makes them suitable for BTE applications [31]. Indeed, pores of 100–150 μm favor new bone formation, bone ingrowth, and capillaries formation, and pores below 50 μm spur the interaction between cells and materials and osteointegration [32].

A silicate glass widely used for the production of scaffolds is 13-93 glass, whose composition is 53SiO_2_–20CaO–6Na_2_O–12K_2_O–5MgO–4P_2_O_5_ (wt %). This glass is characterized by a larger sintering window compared to 45S5 Bioglass^®^, which leads to beneficial viscous flow characteristics and, in turn, to a greater densification [33].

For example, 13-93 scaffolds fabricated by Fu et al. [33] were fully dense, with a smooth surface and pores of 100–500 μm [33]. In addition, the triangular voids resulting from decomposition of the PU foam, typical of Bioglass^®^, were not present in 13-93 scaffolds because they were filled by the facile viscous flow of this glass during sintering [33].

13-93 BG scaffolds with similar morphology were produced by Liu et al. [34]. This resemblance is attributable to the fact that, besides the same glass composition, Liu also used the same slurry composition and sintering temperature used by Fu et al. [33].

In the same study, Liu et al. used the same glass (13-93 BG) also to create scaffolds with oriented morphology by unidirectional freezing of camphene-based suspensions. The oriented scaffolds had a lower porosity (50%) and pore size (50–150 μm) compared to the trabecular one [34].

**Figure 7:**
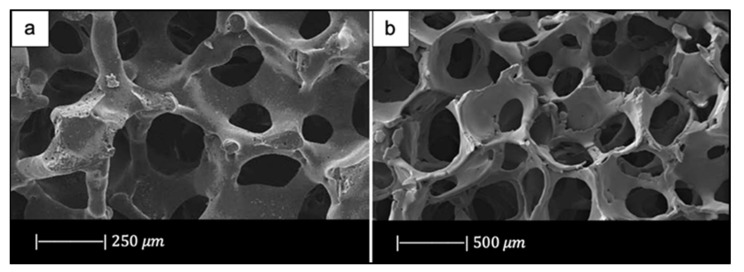
Porous microstructure of (**a**) 13-93 bioactive glass (BG) scaffold [33] (Copyright © 2021 Acta Materialia Inc.) and (**b**) 13-93B3 scaffold [35] (Copyright © 2021, John Wiley and Sons). Figures reproduced from Fu et al. [33,35] with permission.

In another study Fu et al. [35] developed scaffolds of borate glass by replacing all the SiO_2_ in 13-93 with B_2_O_3_. This glass composition, called 13-93B3, fully converts to hydroxyapatite at a rate that is three to four times faster compared to silicate 13-93 scaffolds, thus paving the way for new applications [35]. The replacement of silicon oxide with boron oxide did not lead to significant differences in the final porous structure of the scaffold because the 13-93 and 13-93B3 scaffolds had approximately the same 3D architecture and the same pore size (100–500 μm) (Figure 7) [35]. It should be noted that due to the different glass composition (13-93 and 13-93B3), a different sintering temperature was used; each sintering temperature was appropriate to densify the glass without crystallizing it [33,35].

**Figure 8:**
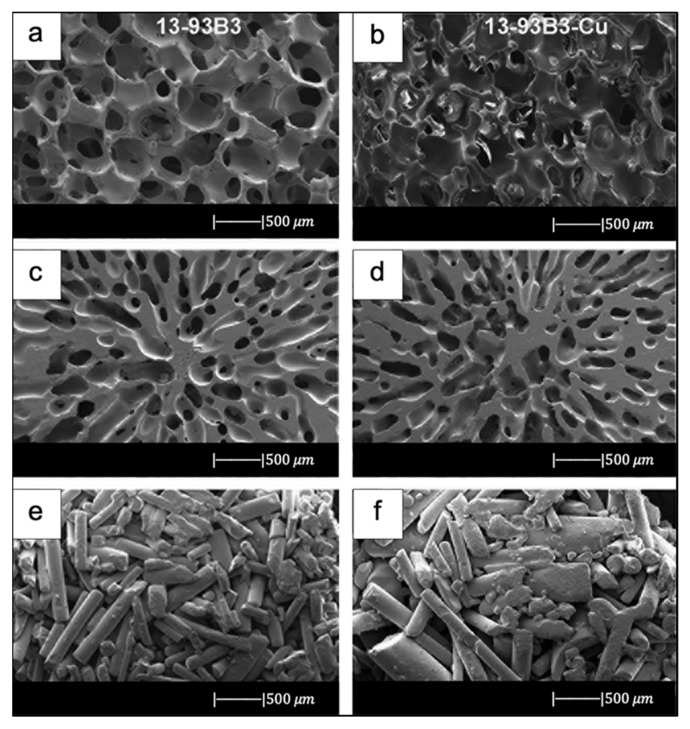
SEM images of 13-93B3 bioactive glass scaffolds (left) and Cu-doped 13-93B3 scaffolds (right) with three different morphologies used in this study: (**a**,**b**) trabecular; (**c**,**d**) oriented, where the pores are oriented in the radial direction; (**e**,**f**) fibrous. Figure reproduced with permission from the authors of [36].

Bi et al. [36] fabricated scaffolds of 13-93B3 glass and Cu-doped 13-93B3 glass with three different microstructures described as trabecular, oriented, and fibrous (Figure 8) [36].

The scaffolds with trabecular morphology were prepared with the polymer foam replication technique, the scaffolds with oriented morphology were fabricated by unidirectional freezing of camphene-based suspensions, and the scaffolds with fibrous morphology were prepared by thermally fusing randomly oriented short fibers [36].

Generally, the architecture and the pore size of the undoped scaffolds were similar to the corresponding doped ones, thus suggesting the independence of morphological properties from the CuO presence (the difference between Figure 8a,b is due to the use of two different SEM devices) [36].

The trabecular scaffolds were characterized by a porosity of 77% ± 5% and pore sizes of 200–400 μm. The fibrous scaffolds showed a porosity of 50% ± 2% and pore sizes of 50–500 μm. The oriented scaffolds exhibited a porosity of 62% ± 5% and columnar pores with diameters between 60 and 200 μm [36]. Therefore, the foam replica method allows us to obtain scaffolds with greater porosity and pore size than scaffolds produced with unidirectional freezing of camphene-based suspensions and thermally fusing randomly oriented short fibers.

13-93B3 scaffolds are limited to the repair of non-loaded bone defects due to their rapid degradation rate. 

This limit is exceeded by 13-93B1 borosilicate glass with the composition 6Na_2_O–8K_2_O–8MgO–22CaO–18B_2_O_3_–36SiO_2_–2P_2_O_5_ (mol %), obtained by replacing one-third of the molar concentration of SiO_2_ in 13-93 glass with B_2_O_3_. This glass degrades and converts faster to hydroxyapatite (HA) than silicate 13-93 glass but slower than borate 13-93B3.

The scaffolds fabricated by Gu et al. [37] with 13-93B1 glass exhibited a trabecular architecture with a porosity of 78% ± 8% and a pore size of 400–650 μm [37].

Since there are no significant differences between the scaffolds produced with 13-93, 13-93B1, and 13-93B3 glass, it is reasonable to assume that the partial or total replacement of SiO_2_ with B_2_O_3_ does not affect the morphological properties of the structure.

By reversing the molar quantities of SiO_2_ and B_2_O_3_ in 13-93B1 glass, the borosilicate glass designated as double alkali borate (D-Alk-B) glass is obtained. Also, this glass produces adequate viscous flow during sintering that allows obtaining dense and smooth struts [38].

For example, Liu et al. [38], by sintering this glass at an appropriate temperature between the Tg and Tx, obtained scaffolds characterized by a very smooth surface and dense struts [38].

The pore size (200–300 μm) was slightly smaller than that typical of scaffolds manufactured using this method [38].

Wang et al., by loading D-Alk-B glass with CuO [39], Fe_3_O_4_ [40], and ZnO [41], respectively, in three different studies, obtained scaffolds with similar morphology. The similarity between these 3D porous structures is due to the fact that they were all manufactured using a sponge of 50 ppi and were sintered at about the same temperature [37,39,40,41]. Furthermore, the fact that these scaffolds all had the same morphology suggested that the doping element did not affect their structure.

In the past several years, mesoporous bioactive glasses have attracted significant attention for bone regeneration due to their high specific surface area, bioactivity, and drug delivery properties [42]. Therefore, MBGs have been used by several researchers to develop scaffolds with the foam replica method for bone tissue engineering applications.

For example, Zhang et al. [43,44] produced MBG scaffolds containing strontium by using co-templates of nonionic block polymer EO20-PO70-EO20 (P123) as mesoporous template and PU sponge as macroporous template [43,44].

These scaffolds exhibited a well-ordered and uniform mesoporous channel configuration with pores of 4.5–5 nm and large pores in the range of 300–500 μm [43,44].

Moreover, it was noted that the presence of strontium caused a reduction in well-ordered mesoporous structures [43,44].

In another study, Sui et al. [45] developed MBG scaffolds using (45)CaCl_2_. The obtained scaffolds had a mesoporous–macroporous cross-linked structure with mesopores of approximately 6.40 nm and macropores in the range of 300–500 μm [45]. 

More recently, the foam replica method was also used to make glass/polymer composite scaffolds. 

The idea of developing composite scaffolds stemmed from the need to obtain porous products with better mechanical properties. 

It is known that bone is a composite material, and the high tensile strength and fracture toughness of bone are largely due to the presence of the collagen fibers [38]. Based on this knowledge, BG scaffolds can be combined with polymers in order to improve their mechanical behavior [46].

For example, Bretcanu et al. [6] produced composite scaffolds by coating 45S5 Bioglass^®^ foams with bacteria-derived poly(3-hydroxy butyrate) (P(3HB)) [6], a thermoplastic polymer characterized by good processability along with suitable biocompatibility and degradation behavior, taking more than 52 weeks for complete degradation in aqueous media.

Firstly, 45S5 Bioglass^®^ scaffolds were fabricated with the foam replica method. During heat treatment up to 1100 °C, the glass crystallized, resulting in glass–ceramic scaffolds. Then the obtained structures were immersed into P(3HB) solution for a fixed time and left to dry [6].

An important result was that the samples maintained a highly porous arrangement with interconnected pores despite the polymer coating [6].

Balasubramanian et al. [47] developed composite scaffolds that imitated the osteochondral region composed of a highly bioactive, porous bone side, and a collagen-containing fibrillar cartilage portion [47].

The foam replica method resulted in highly porous 45S5 Bioglass^®^-based scaffolds similar to the cancellous bone structure. Dip-coating by polycaprolactone (PCL) considerably improved the mechanical behavior of the scaffolds. Electrospinning created a layer of submicrometric collagen–PCL fibrous mesh over the Bioglass^®^ scaffolds [47].

These layers showed alignment of the fibers in between the struts, which is believed to be beneficial for the flattening, elongation, and attachment of chondrocytes [47].

Composite scaffolds were also manufactured with glasses other than 45S5 Bioglass^®^. 

For example, Erol et al. [48] developed boron-containing silicate glass scaffolds coated with alginate cross-linked with copper ions [48].

Alginate formed a uniform coating on the surface of the scaffold without clogging the pores, whose size ranged from 100 to 500 μm [48].

The struts appeared well-densified and the voids resulting from the sponge burn-out were not present. This result suggested that the sintering conditions were correctly chosen, leading to an extensive densification by viscous flow without the formation of crystalline phases [48].

The study of Wu et al. [42] offered an example of a composite scaffold produced by using a mesoporous bioactive glass [42]. This research group used P123 as mesoporous template and PU sponge as macroporous template to produce the scaffold. Then the scaffold was modified with silk solution in order to improve the mechanical properties [42].

The resulting material exhibited a smooth silk film on the surface of the pores. The silk modification improved the continuity and homogeneity of the pore network and did not affect the pore size (200–400 μm) [42].

Recently, a great potential has been attributed to hierarchical systems based on biocompatible mesoporous materials due to the possibility of their providing a unique set of functionalities determined by their peculiar mesoporous texture. The resulting ultra-high specific surface area (SSA) exposed to the physiological environment is responsible for enhanced bioactivity and local release of therapeutic elements [49] such as Si, Ca, Mg, Mn, Cu, Fe, Co, Ga, Ag, and others (for further details on ion release from BG-based scaffolds, the reader can refer to [50]), resulting in a better osteointegration of the implant as well as improved regenerative potential. Moreover, their drug-loading capabilities elicit specific therapeutic effects, i.e., improved osteogenesis and angiogenesis, antibacterial properties [49].

In this regard, Tang et al. [51] produced a trimodal macro/micro/nano-porous scaffold (TMS) by using EO106-PO70-EO106 (F127) as a mesoporous template, methyl cellulose as a microporous template, and PU sponge as a macroporous template. The mesoporous structure was tailored for the recombinant human bone morphogenetic protein-2 (rhBMP-2) entrapment [51].

TMS showed interconnected macropores of 200–500 μm in size, micropores <10 μm, and rhBMP-2-entrapped mesopores of 7.5 nm [51].

### 2.2. Replica of Natural Templates

In recent years, natural structures have been a source of inspiration for biomedical researchers [52,53] in their attempt to improve the mechanical and biological performance of bioactive glass scaffolds supporting bone healing and regeneration. 

#### 2.2.1. Replica of Marine Sponges

The replication of PU foams has created highly porous and interconnected structures that were characterized, however, by low mechanical properties, which limited their suitability for clinical applications in load-bearing sites. As a consequence, marine sponges were investigated as sacrificial templates in order to obtain scaffolds with reduced total porosity and, as a result, increased mechanical properties without the losing the pore interconnectivity required for considerable bone ingrowth [12].

Cunningham et al. [54] were the first to use marine natural sponges as sacrificial templates in place of PU foams for the development of hydroxyapatite-based scaffolds [54]. The obtained scaffolds showed high porosity levels with pores up to 200 μm, which proved to be sufficient for the full integration of the foam in natural tissue [54].

Later, natural marine sponges were used by Boccardi et al. [12] to develop Bioglass^®^-based scaffolds with the replica technique. They used *Spongia Aagaricina* (SA) and *Spongia Lamella* (SL), belonging to the “Elephant Ear” family, harvested from the Indo-Pacific Ocean (Pure Sponges, Solihull, UK) and Mediterranean Sea (Hygan Products Limited, Hartshill, UK), respectively [12]. These sponges, due to the millenarian evolution for water filtration that led to the formation of an interconnected porous structure, are promising materials in the development of bone tissue engineering scaffolds [55]. 

**Figure 9:**
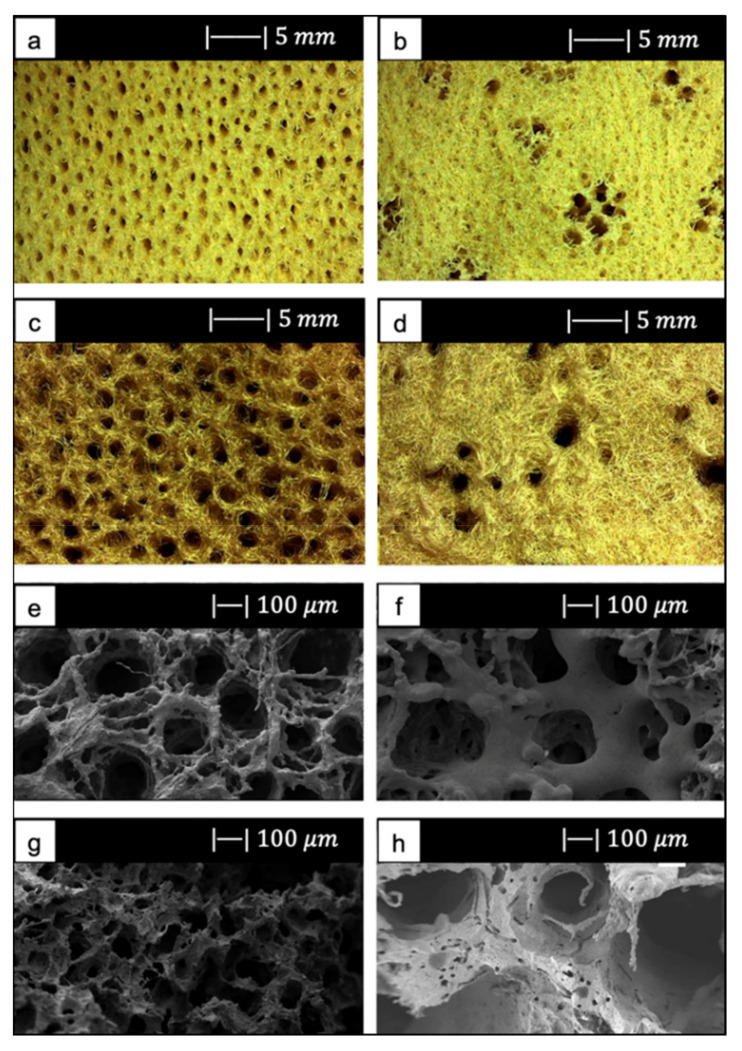
(**a**) Inhalant surfaces and (**b**) exhalant surface of *Spongia Aagaricina* (SA) sponge. (**c**) Inhalant surfaces and (**d**) exhalant surface of *Spongia Lamella* (SL) sponge. (**e**) Inhalant surfaces and (**f**) exhalant surface of SA replica foam (BG-SA). (**g**) Inhalant surfaces and (**h**) exhalant surface of SL replica foam (BG-SL). Figure adapted from Boccardi et al. under the terms of the Creative Commons Attribution License [12].

The SA and SL showed a vase or fan-shaped growth and a surface composed by fine fibers [12]. It was possible to identify two different structures, as reported by Pronzato and Manconi [53]: an inhalant (Figure 9a,c) and an exhalant (Figure 9b,d) surface, depending on the directionality of water flow in the sponges in their natural habitat [12]. Architectural properties of natural marine sponges are summarized in Table 1 [12].

Regarding the scaffold production, the replica technique was used according to the method proposed by Chen et al. [2]. The melt-derived 45S5 Bioglass^®^ powder (particle size ~5 μm) was added to PVA–water solution to obtain the slurry [12]. The SL was immersed in the Bioglass^®^ powder slurry three times, while SA only required two coating cycles. The burn-out and sintering conditions were 400 °C/1 h and 1050 °C/1 h, respectively, and the heating and the cooling down rates were 2 and 5 °C/min, respectively [12].

SEM images of the SA and SL replica foams (BG-SA and BG-SL, respectively) obtained are shown in Figure 9e–h.

SEM images showed that almost all the spaces between the fibers of the marine sponges were filled with the Bioglass^®^ particles, and the macroporous structure was optimally replicated. Indeed, it was still possible to distinguish the inhalant and exhalant surfaces of both kinds of sponge [12].

Small-sized pores connecting the larger pores were observed, creating a highly interconnected porous structure (>99.5%) [12]. 

Overall pore size was in the ranges of 10–600 µm and 10–900 µm, and the average pore size was 215 ± 20 µm and 265 ± 120 µm for the BG-SA and BG-SL, respectively. In particular, the marine sponge foams were characterized by a large number of pores in the range of 0–200 µm, and in the range of 200–500 µm [12].

The presence of a large number of pores in the range of 10–200 µm is fundamental for the complete colonization of the scaffolds by cells, as it also improves the flow of biological fluids inside the porous structure and fosters the complete integration with the surrounding tissue [56,57], whereas pores in the range of 200–500 µm are important for bone integration and neovascularization [56,57].

The total porosity of BG-SL and BG-SA was 76% ± 2% and 68% ± 0.2%, respectively. Hence, the marine sponge foam replicas had a lower total porosity than PU foam replicas, but at the same time these foams had a considerable microporosity, which was almost totally absent from the PU foam replicas [12].

The achievement of improved mechanical properties, which will be discussed later, combined with the high pore interconnectivity and wide pore size distribution, confirmed the choice of using natural marine sponges as porous precursors for fabrication of bone tissue scaffolds [12].

#### 2.2.2. Replica of Bread

Fiume et al. [10] used for the first time stale bread as a sacrificial template for the fabrication of scaffolds for BTE. The choice fell on stale bread because it offers several advantages, such as easy availability, contribution to food waste disposal, and cost effectiveness of the production process [10]. 

They used industrial bread obtained from industrial wastes (Roberto Industria Alimentare S.r.l., Treviso, Italy) [10].

**Figure 10:**
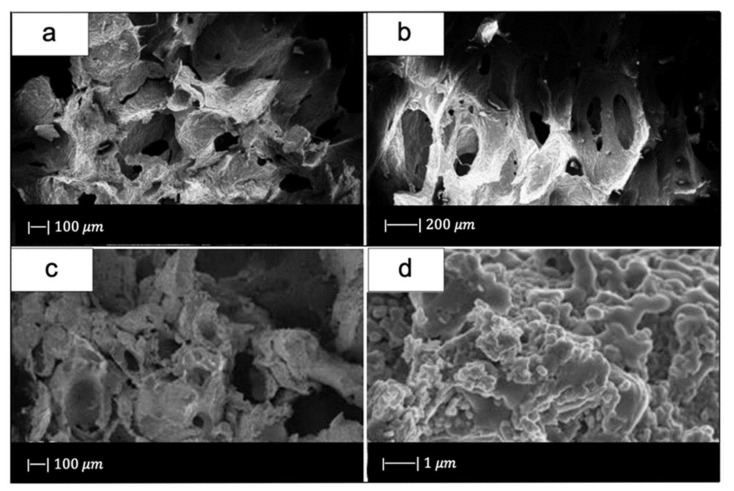
SEM micrographs of (**a**,**b**) bread used, and (**c**,**d**) glass scaffold. Figure reproduced from Fiume et al. [10] under the terms of the Creative Commons Attribution (CC BY) License.

SEM images showed that the industrial bread had a trabecular morphology (Figure 10a,b), which resembled that of cancellous bone [10]. The bread was indeed characterized by pores of regular size and shape, homogeneously distributed and well-interconnected. Both large macropores of 100–300 µm and smaller ones of 10–20 µm were observed [10].

For scaffold production, 47.5B glass with composition 47.5SiO_2_–20CaO–10MgO–2.5P_2_O_5_–10K_2_O–10Na_2_O (mol%) [58], produced with the melt-derived technique, was used as starting material. 

The slurry was prepared by adding the glass to water–PVA solution. The green bodies were thermally treated at 750 °C for 3 h (heating rate 5 °C/min). The sintering temperature was set above the crystallization onset, which was detected at Tx = 700 °C, because of the brittleness of the scaffolds sintered at lower temperatures, as assessed in the early tests [10].

Figure 10c, d shows the SEM images of the obtained scaffolds.

Upon sintering, the sample underwent notable changes both in morphology and in pore size distribution compared to the initial template. However, the morphology of the original bread template was still clearly distinguishable (Figure 10c) [10].

In particular, the densification that occurred during the sintering process caused a decrease in pore size [10]; however, the size was still suitable for TE purposes. Indeed, inter-pore windows of 20–100 µm are sufficient to allow the vascularization of the scaffold [59].

From the images obtained at higher magnifications (Figure 10d), it could be observed in the scaffold the same wrinkled surface as the bread template, which is beneficial for the cell–implant interactions. Indeed, it was found that micrometric roughness on the device surface can foster protein-mediated cell adhesion [60].

In addition, from the SEM images some voids were detected inside the structure. Probably this was due to a poor impregnation of the sacrificial template [10].

The scaffolds had a porosity of 72 ± 1.5 vol %, which lies in the range of spongy bone [10].

Although preliminary results supported the use of stale bread as a template for BTE scaffold manufacturing, the need for a further optimization of the process to enhance its reliability and repeatability is undeniable. The most relevant issue concerning the selected template, in fact, was directly related to the origin of bread porosity within a given volume. It is indeed known that bread crumb porosity is the result of a natural process known as leavening and, thus, it is hardly controllable especially in home-made processes.

#### 2.2.3. Replicas of Vegetal and Fungal Templates

Corn stalks, cattail stems, and mushroom stalks were used as macroporous templates to fabricate HPBG scaffolds [16,61,62].

Jiang et al. [16] implemented the foam replica method in combination with EISA in order to produce hierarchical porous TiO_2_-bioglass scaffolds, using a plant corn stalk as macroporous template and P123 as mesoporous template [16].

They first prepared the precursor solution using P123. Later, the corn stalks were soaked in the precursor solution for 1 day, and then the samples were dried at room temperature for 12 h. The process of dipping and drying was repeated twice for each scaffold. Finally, the samples were thermally treated at 550 °C for 5 h in air to burn out the corn stalk templates [16].

**Figure 11:**
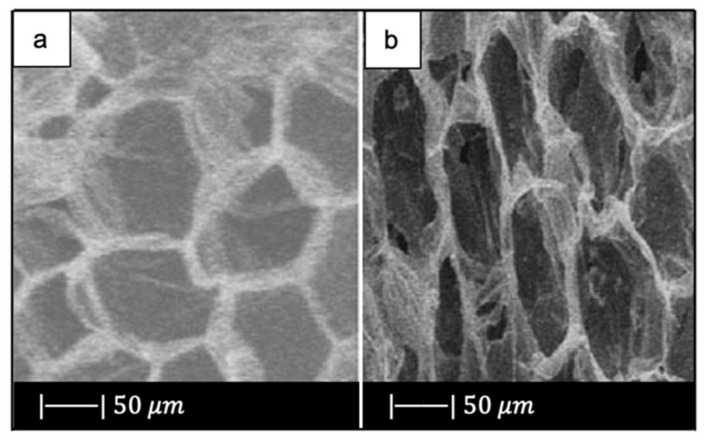
SEM images of (**a**) the corn stalk and (**b**) TiO_2_-glass scaffold. Figure adapted from Jiang et al. [16] with permission (Copyright © 2021, Springer Science Business Media, LLC).

SEM photographs of the corn stalks and the obtained scaffold are given in Figure 11.

As can be seen, the scaffold replicated perfectly the original morphology and structure of the corn stalk, exhibiting a 2D ordered macroporosity characterized by a pore size of about 30–50 µm [16]. 

Macropores >10 µm not only increase the contact surface and flow resistance for cell adhesion, but also carry the blood and the large size matter to a new tissue. In addition, they promote free penetration of cells and intracellular fluid [63].

As regard the nanoporosity, the sample had a pore volume of approximately 0.16 cm^3^/g, a mesopore size of 4.4 nm, and a surface area of 153 m^2^/g [16].

**Figure 12:**
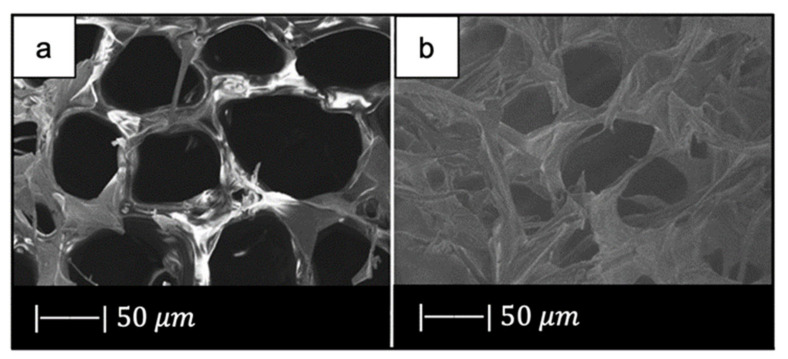
SEM images of (**a**) plant template cattail stalks, and (**b**) the scaffold obtained. Figure reproduced with permission from Ma et al. [61].

Also, Ma et al. [61] used the foam replica method in combination with EISA in order to produce HPBG scaffolds, but a cattail stem with pores between 40 and 60 μm was selected as macroporous template (Figure 12a) [61]. At first, the precursor solution was prepared using P123, then the cattail stem was soaked in the precursor solution for 1 day, and the plant templates were dried at room temperature for 12 h. These processes were repeated twice. After all, the sample was calcined at 550 °C for 6 h [61]. 

SEM images of the obtained HPBG scaffold are given in Figure 12b.

Figure 12b shows that the material approximately copied the macropores structure of cattail stem [61]. Indeed, the scaffold was characterized by the presence of macropores of 30–50 μm in size, where the pore size slightly decreased due to the densification upon high temperature thermal treatment [61].

There is experimental evidence that pores below 50 μm stimulate the interaction between cells and materials, and osteointegration [32].

The hierarchical porous glass had a pore volume of 0.137 cm^3^/g, a mesopore size of 3.86 nm, and a surface area of 154.22 m^2^/g [61]. 

Thus, the HPBG obtained by Ma et al. [61], with both macroporous and mesoporous structures, was found to be suitable for bone tissue engineering and drug delivery [61].

**Figure 13:**
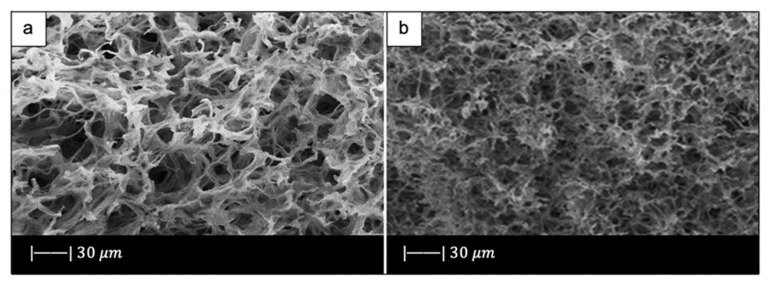
SEM images of (**a**) mycelium and (**b**) bioactive scaffold obtained by replicating mushroom stalk (MRBG). Figure adapted from Han et al. [62] with permission (Copyright © 2021, Springer Science Business Media New York).

Han et al. [62] fabricated hierarchical porous bioactive glass (system SiO_2_–CaO–P_2_O_5_) scaffolds by using a dual-template method [62]. Mushroom stalk was used as the macroporous template, and P123 was used as the mesoporous template. The choice of the macroporous template fell on the mushroom stalk because of its unique three-dimensional mycelium and high pore volume. Mycelium is the vegetative part of mushrooms, composed of a mass of branching, thread-like hyphae. It is characterized by a 3D porous structure of 20–40 µm in size, as shown in Figure 13a [62]. As regards the scaffolds production, Han et al. [62] prepared a mesoporous bioactive glass precursor (MBG-p) solution using P123 as mesoporous template. The mushroom stalks were soaked in MBG-p solution for 1 day, and then the samples were dried at room temperature for 12 h. This process of dipping and drying was repeated twice for each scaffold. Finally, the samples were thermally treated at 550 °C for 5 h in air to burn out the mushroom stalk templates. The obtained hierarchical porous materials were named MRBG [62].

Figure 13b shows a representative SEM image of MRBG [62].

The obtained scaffold showed the reticulate structure of the vegetal template with a pore size of 10–30 µm. Therefore, the three-dimensional structure of the mycelium was replicated perfectly. However, the MRBG showed decreased diameter compared to mycelium due to the high temperature treatment [62].

As regards the nanoporous texture, the MRBG had a pore volume of approximately 0.14 cm^3^/g, a mesopore size of 3.4 nm, and a surface area of 107.28 m^2^/g [62].

Due to both the macroporous and mesoporous structures, the MRBG exhibited good biomineralization, cell attachment, and controlled drug delivery ability. These aspects made the obtained scaffold a promising material for tissue regeneration [62,63].

#### 2.2.4. Replica of Demineralized Bone Matrix

Using bone matrix as sacrificial template can be a valuable strategy to replicate the most peculiar morphological features of trabecular bone [64]. 

The use of calcined bone matrix allows accurately duplicating the macroporous bone architecture, while the reproduction of the fine microstructure is partially hindered by intrinsic technological limitations (e.g., maximum achievable resolution with available methods) [65,66]. 

In contrast, demineralized bone matrix (DBM) is an excellent sacrificial template because it also retains the ordered microporous structure of natural bone thanks to the removal of well-assembled hydroxyapatite nanoclusters from the bone matrix. In addition, the intrinsic elasticity of the organic matrix facilitates the infiltration of the ceramic/glass suspensions, thus allowing a complete impregnation of the template [64].

Demineralized cancellous bone matrix was used by Xia et al. [64] as a sacrificial template to developed a biomorphic bioactive glass–gelatin composite scaffold having both structure and mechanical behavior similar to cancellous bone [64].

As starting materials for the scaffold manufacture, 58-S sol–gel glass and gelatin (Sinopharm, China) were used [67]. 

In particular, bones from bovine femoral distal condyles and from bovine femoral heads were selected, and DBM was prepared according to published procedures [65,66]. 

The demineralized bone matrix templates were soaked in the glass slurry and the obtained samples were calcined at 700 °C for 3 h at a heating rate of 1 °C/min to remove the organic matrix. Then the obtained bioactive glass scaffold was immersed in gelatin solution to allow the gelatin solution to enter into the gaps in the pore walls of the BG scaffold. The obtained composite scaffold was then dried at room temperature [64].

**Figure 14:**
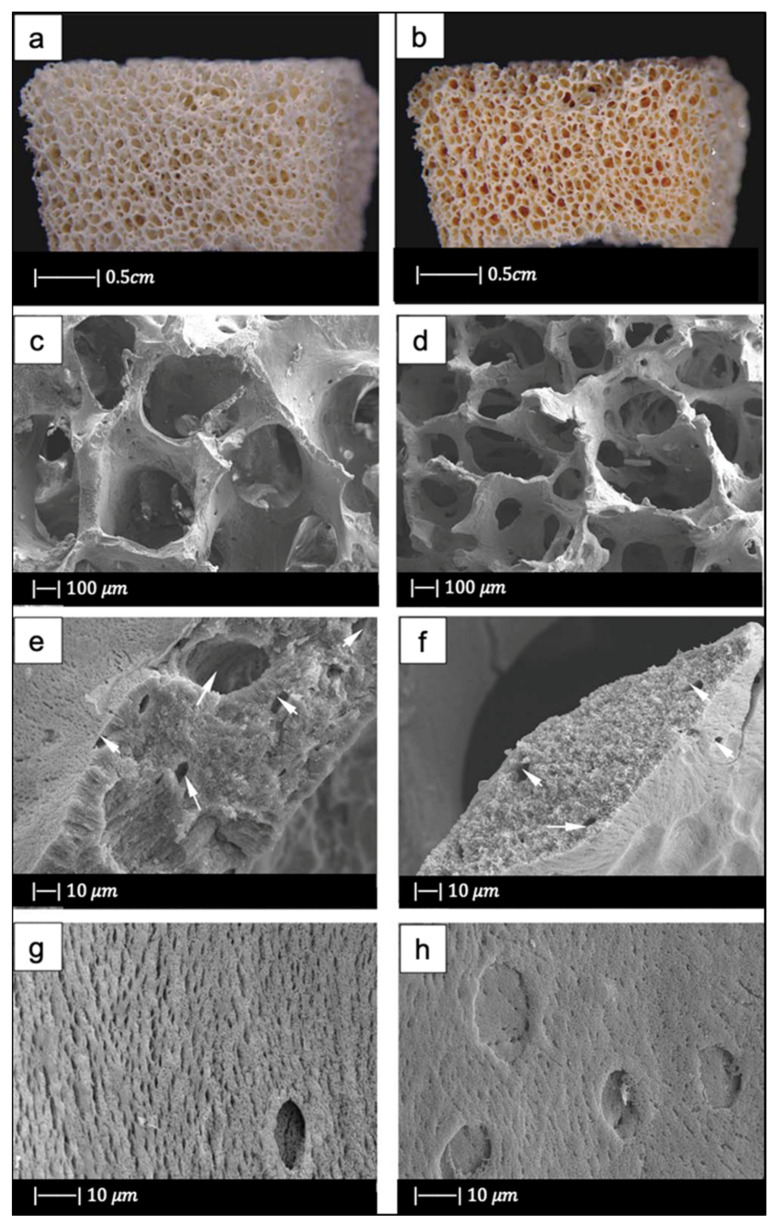
(**a**) Bioactive glass scaffold. (**b**) Bioactive glass/gelatin composite scaffold. SEM images of (**c**,**e**,**g**) biomimetic bioactive glass scaffold, and (**d**,**f**,**h**) natural calcined cancellous bone. Figure adapted from Xia et al. with permission [64] (Copyright © 2021, John Wiley and Sons).

Figure 14 shows the optical images of a bioactive glass scaffold and its corresponding composite scaffold, as well as SEM images of a biomimetic bioactive glass scaffold and natural calcined spongy bone [64].

Both the BG scaffold and the bioactive glass/gelatin composite scaffold were characterized by high porosity and good pore connection (Figure 14a,b) [64].

It was noticed that the BG scaffold had macroscopic and microscopic structures similar to calcined cancellous bone (Figure 14c–h) [64]. 

The macrostructure of the bioactive glass scaffold (Figure 14c) was characterized by pores of 300–700 μm and an excellent pore interconnection [64]. 

Figure 14e shows that the bioactive glass scaffold perfectly replicated the microporous structure of the calcined spongy bone matrix (Figure 14f) [64]. 

As can be seen from Figure 14g, bioactive glass particles in the pore walls had a well-oriented arrangement. In addition, between the glass particle bunches, ordered bunches of holes could be observed. Similar holes, which connected the macropores on the pore wall, were also present in the calcined spongy bone (Figure 14h) [64].

The porosities of DBM, calcined spongy bone, and the BG scaffold were 77.1% ± 2.1%, 86.6% ± 1.5%, and 89.3% ± 2.0%, respectively. The porosity of bioactive glass/gelatin composite scaffold was around 88% [64]. These porosities lie in the porosity range of the spongy bone, which is between 50 and 95% [68].

Therefore, by using DBM as a template, a biomorphic scaffold with highly porous macrostructure and fine microstructure was produced. The most interesting aspect of the composite scaffold was its excellent mechanical properties, which will be discussed later [64].

## 3. Mechanical Properties of Scaffolds Produced with the Foam Replication Technique

Just like the morphological features, the mechanical properties can also be significantly influenced by each specific sacrificial template, as the compressive strength of the scaffold is directly related to porosity level and the organization of struts in the three-dimensional space. 

Other factors that can indirectly affect the mechanical performances of the scaffold are the composition of both the initial glass and the slurry, the number of consecutive immersions, and the duration of each single immersion.

In addition, the mechanical performances are also affected by process parameters, such as the sintering temperature and the heating rate.

Mechanical properties of scaffolds for BTE applications are usually evaluated in terms of the stress–strain curve as well as compressive strength. 

**Figure 15:**
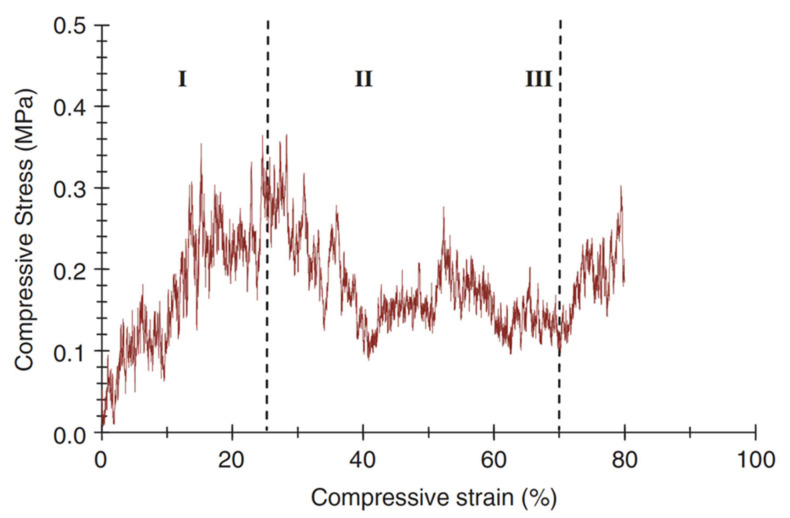
A typical compressive stress–strain curve of the 45S5 Bioglass^®^-based foams sintered at 1000 °C for 1 h. (The porosity of the foam was 91%.) Figure reproduced with permission from Chen et al. [2] (Copyright © 2021 Elsevier Ltd.).

A typical compressive stress–strain curve of a scaffold fabricated with the foam replica method is shown in Figure 15 [2].

It can be observed that the stress–strain curve is “jagged”, i.e., characterized by the presence of multiple peaks typical of cellular ceramic materials under compression conditions. As soon as the load is applied, the scaffold tends to crack in thin struts at stress-concentrating sites, causing a temporary decline of apparent stress. However, the overall structure still has the ability to bear increasing loads, thus determining the increase in stress values [2].

Three different regions of the stress–strain curve can be identified:

- Stage I: the stress–strain curve shows a positive slope until a maximum stress is reached.
- Stage II: the maximum stress reached in stage I causes the thick struts of the foam to fracture, leading to a negative slope in stage II.
- Stage III: fractured foam densifies as stress increases, showing the typical behavior of glass and glass–ceramic scaffold under the action of compressive loads [2].

When designing a scaffold for load-bearing applications, a balance between porosity and the mechanical features must be achieved: while a highly interconnected porous structure with a minimum porosity value of 50 vol % is required for the scaffold to fully integrate once implanted, a proper mechanical integrity has to be ensured for the whole duration of the treatment, until complete healing. This is actually not simple, as strength and stiffness progressively decrease when the volume fraction of porosity increases [69,70]. 

Mechanical properties, pore size, and porosity of several bioactive glass scaffolds produced with the foam replica method are summarized in Table 2.

As can be seen, most of the scaffolds produced with the foam replica method have porosity levels and a compressive strength comparable to those of spongy bone and thus are potentially suitable to be used in BTE applications. Indeed, the reported scaffolds show a level of porosity between 56 and 94%, which fall within the typical range of spongy bone (50–95 vol %). In addition, their compressive strength was found to be between 0.16 and 18 MPa, again within the typical values reported for human trabecular bone (0.1–16 MPa). 

The low compressive strength exhibited by the 45S5 Bioglass^®^ scaffolds developed by Chen [2] was due to the highly narrow sintering window of this glass [25]. Indeed, 45S5 Bioglass^®^ suffers from limited viscous flow and crystallizes prior to densification [25]. Accordingly, the voids resulting from the burn-out of the PU foam are not easily filled and remain as triangular-shaped pores in the struts; these pores cause a decrease in the strength of the scaffold [4].

In addition, it should be considered that the 45S5 Bioglass^®^ scaffolds developed by Chen [2] are the most porous of all the scaffolds analyzed in Table 2. As already mentioned, generally a high porosity is responsible for low mechanical performance [70].

Since it was found that the compressive strength of an HA scaffold considerably increases in vivo due to tissue ingrowth, developing a scaffold with a mechanical strength equal to bone might not be required because cultured cells on the scaffold and new tissue formation in vitro form a bio-composite and considerably increase the time-dependent strength of the foam [71].

However, an ideal scaffold should have at least an appropriate strength and fracture toughness in order to be handled for tissue engineering applications and to ensure an initial stability of the implant. In view of this, 45S5 Bioglass^®^-based scaffolds possessed a suitable mechanical competence [2].

Baino et al. [27] obtained more resistant 45S5 Bioglass^®^-scaffolds compared to those fabricated by Chen [2]. They achieved this result by sintering the glass at a higher temperature (1180 °C) [27] than the one used by Chen (1000 °C) [2].

An increment of sintering temperature led to an increase in mechanical properties for two reasons: (1) sintering at a higher temperature involves a greater volumetric shrinkage of the scaffold, entailing in turn a lower porosity in the final structure; (2) by using a higher sintering temperature, an improved densification of scaffold trabeculae is obtained [27].

Despite the high sintering temperature used, these scaffolds had a porosity level (70%) sufficient for BTE applications [27].

Equally resistant 45S5 Bioglass^®^ scaffolds were obtained from the marine sponge replicas. Indeed, the reduced porosity of the natural marine sponges made it possible to create scaffolds with reduced porosity, and as a result, high compressive strength [12].

Moreover, natural marine sponges were characterized by interconnected porous structures, which resulted in 45S5 Bioglass^®^-based scaffolds with high pore interconnectivity and wide pore size distribution [12]. 

Compared to marine sponges, the use of stale bread as a sacrificial template led to a decrease in mechanical properties; however, the compressive strength of bread-templated scaffolds was about two times higher than that of the PU-derived 45S5 Bioglass^®^ scaffolds fabricated by Chen et al. [2] and almost comparable to those produced by Baino and coworkers using ICEL2 as the basic glass system.

CEL2-derived scaffolds fabricated by Baino et al. [27] had twice the mechanical strength of the Bioglass^®^-derived scaffolds used as control in the study. 

The different compressive strength cannot be due only to the almost negligible decrease of porosity; it was attributed mainly to the different sintering windows of the two types of glass [27]. Indeed, as already stated, due to the narrow sintering window, Bioglass^®^ crystallized before the occurrence of a significant viscous flow sintering; while, in CEL2, glass the viscous flow sintering began at Tg and was fully completed when crystallization began. Therefore, the CEL2 glass reached a higher densification compared to Bioglass^®,^ and as a result, it was more resistant [27].

In addition, the different mechanical behavior of the two types of scaffolds was also due to the different crystalline phases in the materials. The densities of CEL2 scaffold crystalline phases were higher in comparison to that of the Bioglass^®^ scaffold. Because the higher the density of a material, the higher is its mechanical strength, the crystalline phases of CEL2 scaffolds gave the structure higher mechanical strength in comparison to Bioglass^®^ scaffolds [27]. Moreover, subsequent studies demonstrated the high dependency of CEL2-based scaffolds’ mechanical properties on the sintering temperature [27,29].

Moreover, CEL2 scaffolds sintered at lower temperatures were characterized by the presence of a large number of pores that reduced the resistant section and led to the collapse of the structure in some inner parts of the scaffold, probably attributable to a deficient glass coating in the inner part of the sponge [29]. 

Scaffolds based on ICEL2 [9], which is a phosphate glass obtained by modifying the chemical composition of CEL2, showed a lower compressive strength than CEL2 scaffolds [27].

This can be attributed both to the different pore contents, which were higher in ICEL2 scaffolds than in CEL2 scaffolds, and to a lower degree of sintering reached in ICEL2 scaffolds with respect to CEL2 scaffolds due to the narrower sintering window [9]. 

The first scaffolds produced of calcium phosphate glass had higher compressive strength [3] compared to ICEL2 scaffolds [9]. This was attributed to the repeated slurry coating/sintering process, which led to thickening of the struts, and to the use of a lower heating rate. Indeed, as mentioned above, the use of a heating rate lower than 1 °C/min involved removing the sponge only after the formation of necks between the particles and the creation of sufficient binding strength between the particles.

In addition, phosphate glass scaffolds exhibited a dense structure without voids and cracks, which was responsible for high mechanical properties [3].

SCNA and 1393 silicate glasses also showed suitable thermal behavior for obtaining mechanically performing scaffolds.

SCNA-based scaffolds having the mechanical suitability for load-bearing bone repair were successfully produced by Baino and Vitale-Brovarone [31]. The elastic modulus of the obtained scaffolds, 380 ± 172 MPa, was within the range assessed for spongy bone (50-500 MPa), and the compressive strength was above the range of the cancellous bone compressive strength; however, a value higher than that of the bone may cause bone resorption [31].

The high mechanical behavior is attributable to the suitable sintering temperature used, which led to a great densification of scaffold trabeculae and a high volumetric shrinkage of the structure, resulting in a reduced porosity [31]. The porosity value of the final scaffold was, however, above the minimum value required (50%) for tissue ingrowth [32,72]. 

13-93-basedscaffolds fabricated by Fu et al. [33] had approximately the same porosity of 45S5 Bioglass^®^ scaffolds but a compressive strength of almost 20 times the value of the latter. This difference in strength was due mostly to the different sintering windows of the two glasses [4]. Indeed, as already mentioned, 13-93 glass is characterized by a larger sintering window in comparison to 45S5 Bioglass^®^, which involves beneficial viscous flow features and, in turn, a higher densification, thus causing an increase in the compressive strength of the scaffold [33].

Scaffolds of 13-93B3 glass, which are obtained by replacing all the SiO_2_ in 13-93 with B_2_O_3_, had roughly the same porosity as 13-93 scaffolds but only half the compression strength. The decrease in compressive strength is attributable to the presence of boron oxide. Indeed, it was found that the compressive strength decreased with the increasing content of boron oxide [35].

In support of this, the D-Alk-B glass scaffolds produced by Liu et al. [38], composed of a smaller amount of B_2_O_3_ than 13-93B3, exhibited a higher compressive strength than 13-93B3 scaffolds [35], and a lower compressive strength compared to 13-93 glass scaffolds [33].

The high mechanical behavior of the D-Alk-B glass scaffolds was reached thanks also to thicker coated green bodies, thus switching the load-bearing units from the trabeculae to the pore walls [38].

The different mechanical behavior between glass (or glass–ceramic) scaffolds and bone is mainly due to the fact that the glass is an inorganic material, while bone is a composite material, composed of inorganic and organic substances. Therefore, a composite scaffold made of bioactive glass and polymer, having intermediate properties between those of glass and polymer, should better imitate the behavior of bone. Natural polymers are usually preferred to synthetic ones as they do not release any toxic chemical species in the biological environment upon degradation. Despite this, natural polymers typically show lower stability in terms of physical and mechanical properties compared to synthetic ones and suffer from poor processability, which sometimes makes the production of composite scaffolds a true challenge. Additionally, natural polymers can be affected by variable final properties according to their source, risk of contamination caused by the presence of microbes, uncontrolled water uptake, and unpredictable degradation rates. 

As a result, an appropriate balance between glass content, good mechanical properties, and degradation kinetics should be carefully considered. Indeed, the hydroxycarbonate apatite on the surface of bioactive glasses has a stimulatory effect on the differentiation of stem cells toward osteoblast cells, especially when therapeutic ions are introduced [73]. 

Several research groups have developed bioactive glass/polymer composite scaffolds [74]. For example, Bretcanu et al. [6] produced 45S5 Bioglass^®^/P(3HB) composite scaffolds. P(3HB) is a natural thermoplastic polymer produced by several types of microorganisms [75]. The work of fracture of the scaffolds is greatly increased after coating with P(3HB). It is believed that the polymer layer covers and fills the microcracks located on the surfaces of the struts, improving the mechanical stability of the scaffold [76]. Furthermore, the polymer coating does not influence the pores’ interconnectivity [6].

In addition to P(3HB) coating, PCL coating increased 45S5 Bioglass^®^ mechanical properties [40]. According to Hum et al. [77] PCL coating increases the scaffold stiffness by 58% in comparison with uncoated scaffolds [77]. In addition, it was found that PCL coating did not clog the micropores that are necessary for effective cell seeding and bone tissue ingrowth [47].

Polymeric coatings also improved the mechanical behavior of scaffolds obtained with glass other than 45S5 Bioglass^®^. For example, coating with alginate cross-linked with copper ions increased the compressive strength of boron-containing silicate glass scaffolds produced by Erol et al. [48]. The alginate layer covered the struts and filled microcracks on the struts’ surface, increasing the mechanical stability of the scaffolds [48]. This agrees with the research of Mouriño et al. [78] and with a previous investigation in the field of polymer-coated porous ceramics [76,79]. In addition, it was observed that the compressive strength values increased with copper content. This phenomenon could be due to increased cross-linking density; however, this hypothesis was not demonstrated [48]. 

Apart from alginate, gelatin is also highly effective in improving the mechanical properties of bioactive glass scaffolds. Indeed, 58S–gelatin composite scaffolds produced by Xia et al. [64] using DBM as a sacrificial template, showed a low strength before adding gelatin. After compounding the BG scaffolds with gelatin, the compressive strength increased considerably. More specifically, the compressive strength doubled after adding 1 wt % gelatin solution. By increasing the concentration of gelatin, the compressive strength continued to increase. Once the gelatin concentration became higher than 10 wt %, the average compressive strength was close to 5 MPa, which is about 30 times the strength of the scaffold before compounding with gelatin [64]. In addition, the gelatin gave the scaffolds a high resistance to deformation and an excellent shape restoration property. The potential of these glass–gelatin composite scaffolds is that the mechanical properties of the scaffolds greatly increased, although the porosity was the same as before the addition of gelatin [64].

Since generally polymers increase the mechanical properties of the scaffolds while preserving their porosity, they have proved useful in the field of mesoporous bioactive glass scaffolds. As mentioned above, pure MBG scaffolds have many advantages, such as high bioactivity and drug-loading ability. However, their applicability is limited by their inherent brittleness, which results in a non-continuous and collapsed pore network and low mechanical strength. For example, Wu et al. [42] used silk to improve the mechanical behavior of MBG scaffolds. The addition of silk greatly increased mechanical strength, placing it within the range of the compressive strength of spongy bone, and maintained high porosity [42].

There are two possible reasons why silk addition enhanced the mechanical behavior of the MBG scaffolds: (1) silk may have created a more uniform and continuous pore network within the MBG scaffolds, which led to higher compression strength; or (2) silk, which has higher mechanical strength than any other traditional polymer [80], may have formed an intertexture within the MBG scaffolds, connecting the inorganic phases together and strengthening the scaffolds [79].

## 4. Mass Transport Properties

In tissue engineering, the use of scaffolds is mainly aimed at providing a suitable environment to favor cell adhesion, proliferation, migration, and thus, the formation of new tissues [81]. Specifically, scaffold microstructure is crucial to proper cell infiltration within the whole 3D volume as well as tissue vascularization, nutrient supply, and waste removal [82]. In particular, several studies have reported that an appropriate nutrient transport through the scaffold is required for cell cultures [83,84,85,86], in both static and dynamic conditions. Accordingly, the ability of the scaffolds to be permeated by nutrients and metabolites, i.e., the easiness with which biological species and fluids migrate through the scaffold [87], strongly affects TE processes [88,89,90,91].

Mass transport phenomena through scaffolds occur at different scales, including the molecular level (nanoscale), the single-pore dimension level (microscale), and the whole-sample level (macroscale) [92]. Typically, scaffold microstructure is investigated in terms of pore size and distribution, porosity, pore tortuosity, and surface area. However, considering these parameters can sometimes lead to confusion when characterizing the mass transport properties of scaffolds [93]. For example, scaffolds with high porosity are believed to have better mass transport properties when compared to those with lower porosity; however, it was also found that scaffolds characterized by the same porosity do not necessarily exhibit similar transport of nutrients [94]. 

Moreover, it is worth mentioning that an uncontrolled micro architecture resulting in heterogeneous pore size and distribution as well as the presence of isolated and dead pores may determine an uneven distribution of cells within the volume, thus hindering cellular penetration and new matrix formation [95]. Furthermore, determining pore tortuosity is usually a challenging issue due to clear difficulties in measuring the real route length of porous microchannels [93]. On the other hand, the assessment of intrinsic permeability is quite easy and allows appraising mass and species transport through the scaffold and defining its topological characteristics [82]. 

Permeability quantifies the ability of a porous structure to be crossed by a fluid when subjected to a pressure gradient. Thus, in tissue engineering applications, permeability is a useful parameter to qualitatively evaluate the nutrient flow to cells within the scaffolds. Several studies have stated that cell growth within 3D scaffolds depends on how well nutrients can permeate through the porous medium during the cell culture process [83,84,85] or after implantation. 

Permeability is a macroscale property that significantly influences biophysical stimuli at the microscale due to the effect of fluid flowing through the scaffold during physiological mechanical loading [90]. In addition, permeability influences the pressure magnitude and shear forces within a structure, denoted as potential stimuli for cellular differentiation or functional adjustment [96,97,98] for the effectiveness of cell seeding [99,100] and for the formation of new tissue in vivo [101]. Furthermore, permeability affects the degradation rate of biodegradable scaffolds [102,103]. 

Permeability is commonly evaluated by Darcy’s law. At the macroscale, the Darcy flow transport model defines the fluid flow through a porous structure as the consequence of a proportionality between the fluid velocity and the applied pressure difference (forcing term) (Equation (1)) [104]:

(1) $$-\frac{\partial P}{\partial x}=\frac{\mu }{k}\;U$$

where:

*x*: flow direction,
*∂P/∂x*: pressure gradient,
*μ*: fluid dynamic viscosity,
*k*: intrinsic permeability of the porous medium.

The intrinsic permeability depends entirely on the scaffold pore structure [104]. In particular, the intrinsic permeability is a function of the pore morphology, interconnection, and pore size, as well as overall porosity [105]. In the first approximation, intrinsic permeability is directly proportional to the porosity of the scaffold. However, scaffolds characterized by the same porosity may have different values of intrinsic permeability because of differences in pore size, morphology, and architecture [105]. 

It is also worth mentioning that with increasing porosity, the apparent scaffold stiffness decreases according to the square of porosity [106]. Thus, a scaffold should have a suitable porosity allowing sufficiently high permeability for waste removal and nutrient supply and appropriate stiffness to bear the loads conveyed to the scaffold from the surrounding healthy bone [105]. 

In addition, cell attachment and migration to the scaffold surface are influenced by the bulk biomaterial stiffness [107] and the available specific surface. The specific surface is in turn related to permeability as a function of the microstructural design of the scaffold and porosity, which determine the overall permeability, as stated above [105]. Therefore, in the scaffold design, it is crucial to consider permeability, since it is an important parameter that affects others and, accordingly, the final success of the scaffold [105].

In the light of what has been said, in order to quantitatively evaluate the resemblance of a BG scaffold pore interconnectivity with that of spongy bone, it is instructive to compare the permeability values of the two structures [105]. 

Regarding the spongy bovine bone, values between *k* = 2 × 10^−9^ and 9.5 × 10^−9^ m^2^ were reported [108] in the range of porosities 80–90%. Moreover, values of *k* = 7.22 × 10^−9^ and 5.13 × 10^−9^ m^2^ were presented for human spongy bone of the vertebra body and proximal femur [109]. 

Ochoa et al. [105] measured the permeability of porous Bioglass^®^ scaffolds produced with the foam replica method. These scaffolds were characterized by a porosity of 90–95% [95]. The assays were carried out in apposite equipment designed for permeability tests to measure intrinsic permeability in the Darcy’s linear region [105]. The measured intrinsic permeability, *k* = 1.96 × 10^−9^ m^2^, fell within the range of the reported experimental data for human cancellous bone. This confirmed that Bioglass^®^ scaffolds, produced with the foam replica method, had transport properties and pore structure similar to those of spongy bone, and thereby represented interesting artificial extracellular matrix structures for the BTE applications [105].

Recently, Fiume et al. [110] proposed a multidisciplinary approach for the accurate assessment of the overall set of descriptive parameters of the porous microstructure of foam-replicated bioactive glass and glass–ceramic scaffolds for bone regeneration. 

Mathematical modeling based on the Forchheimer theory and supported by micro-computed tomography imaging was implemented to selectively investigate the effect of different pore features on intrinsic permeability, experimentally determined by laminar airflow alternating pressure wave drop measurements. Within the considered temperature range, permeability values were comparable to those reported in literature for human trabecular bone [109], thus confirming the potential suitability of the scaffolds to support cell migration and mass transport phenomena through the whole volume of the graft. 

Interestingly, a clear dependence of glass characteristic temperatures and scaffold permeability variation upon different sintering treatments was observed, thus suggesting the possibility of extending the described model to a wider range of glass-based porous struts, even outside the biomedical field [110].

## 5. In Vivo Studies

Nowadays, in vitro and ex vivo studies are considered an essential step that has the purpose of providing a preliminary evaluation of the biological response induced by the material [17,111]. However, despite their undeniable scientific and ethical importance, in vitro and ex vivo tests, conducted outside of a living organism and in an artificial environment [111], are intrinsically limited by the reduced complexity of the testing conditions, able only to approximate the performance of the scaffolds in vivo.

As an example, during in vitro experiments, immune or inflammatory responses do not take place, as well as the cascade of events arising from in vivo implantation, e.g., the interaction with blood components, clot formation, vascularization, and cell recruitment for wound-healing processes [112]. 

In vitro toxicity levels of materials are generally overstated, and since the cultured cells have a relatively short life span, the in vitro studies allow evaluating only the acute toxicity [113]. Moreover, in vitro studies are commonly performed in 2D, on planar or curved surfaces, which do not resemble the 3D surroundings typical of biological tissues and organs [17]. For all these reasons, in vitro assessment of biomaterials has questionable clinical relevance [17]. 

On the other hand, ex vivo models are more complex in terms of cell diversity, and therefore closer to in vivo conditions, but exhibit lower reactivity upon exposure to a specific treatment or stress [111]. 

In vivo animal studies enable us to understand and assess material’s performance in the complex physiological environment and represent an inevitable stage before clinical trials. They allow us to evaluate the biomaterials under various loading conditions, for long periods of time, in several tissue qualities (e.g., healthy or osteopenic bone) and age [17]. However, in vivo animal studies also have limits; for example, an animal model that has physiological and pathophysiological similarities with humans must be chosen, and the operation must be controllable. In addition, the implant size, number of implants per animal, and test duration should follow the international standards. Other limits include animal availability, costs of purchase and care, the animal’s ability to withstand testing, and social acceptability [114].

**Figure 16:**
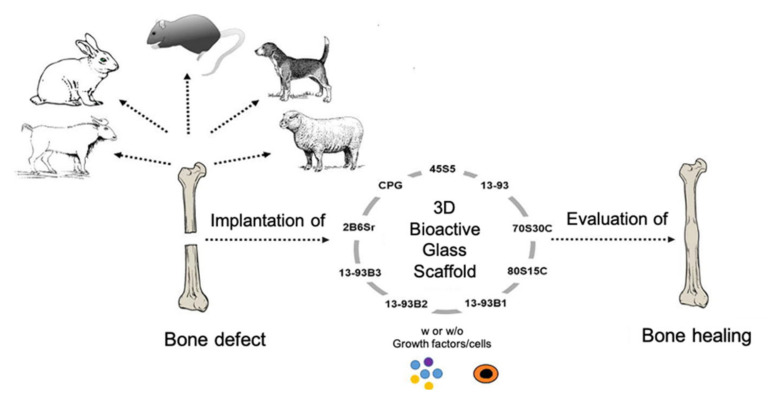
Schematization of in vivo studies on bioactive glass scaffolds. Figure reproduced with permission from El-Rashidy [17].

Figure 16 provides a schematization of in vivo studies on bioactive glass scaffolds.

The bone defect model chosen as implant site for the scaffold should be a good representation of the designed clinical application. Bone defect models include calvarial, long bone, or maxillofacial defects; they can be classified into non-critical and critical-sized bone defects, and are used to test osteocompatibility and osteogenesis, respectively. The calvarial defect is generally used as a non-load-bearing model for studying scaffolds with mechanical properties inferior to bone, while load-bearing long bone defect models (e.g., femur, tibia, radius, and humerus) are commonly used for investigating scaffolds with properties similar to bone [115].

In vivo studies performed on BG-based scaffolds produced with the foam replication technique were comprehensively reviewed by El-Rashidy et al. [17]. 

Most of the case studies reported below are based on calvarial models of rats. Indeed, the rat is the first choice for in vivo testing of biomaterials for BTE due to its small size and easy handling and housing [114].

**Figure 17:**
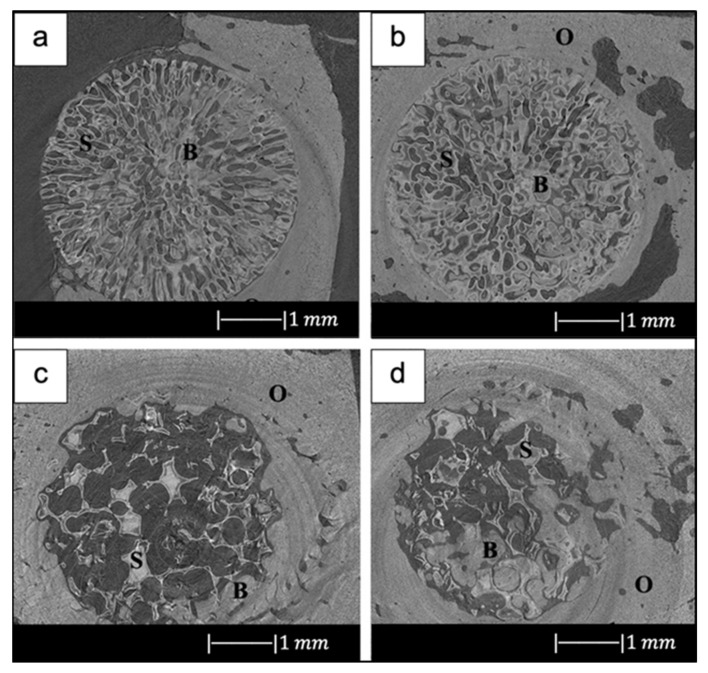
Synchrotron micro-computed X-ray tomography images of 13-93 BG oriented scaffold after implantation for (**a**) 12 weeks and (**b**) 24 weeks, and 13-93 BG trabecular scaffold after implantation for (**c**) 12 weeks and (**d**) 24 weeks. The distribution of old bone (O), new bone (B), and the bioactive glass scaffold (S) is given. Figure reproduced with permission from Liu et al. [34].

For example, in the study of Liu et al. [34], 13-93 BG scaffolds with two different microstructures (oriented and trabecular) were compared for their capacity to regenerate bone and osteointegration in a rat calvarial defect model [34]. Both the oriented and trabecular scaffolds were able to support bone regeneration in rat calvarial defects, but the microstructure greatly affected the way in which new bone infiltrated the scaffold and the integration between the scaffolds and the host bone (Figure 17) [34]. Twelve weeks after implantation, new bone formed principally on the dural (bottom) side of the oriented scaffolds, and only a small amount of bone ingrowth into the periphery (edge) of the scaffolds was detected. At 24 weeks, total bone regeneration was enhanced, as well as bone infiltration into the periphery of the scaffolds and integration with host bone (Figure 17a,b) [34], while the trabecular scaffolds exhibited bone regeneration mainly into the implant periphery and greater integration with the host bone than the oriented scaffolds at 12 weeks (Figure 17c,d) [34]. The different bone infiltration in the two kinds of scaffolds (the dural side vs. at the periphery) may be attributed to the different architecture of the scaffolds [34].

It was found that besides porosity and pore size, other factors, such as pore interconnectivity, the size of the aperture between close pores, permeability and microstructural anisotropy of the structure, influence bone infiltration [116,117]. Indeed, even though the trabecular scaffolds had a higher porosity and pore size, the more tortuous pore channels may have constrained bone formation to the periphery. Although the oriented scaffolds had a lower porosity and pore size, the pores were more connected, and the pore channels were less tortuous, which may have fostered bone ingrowth inside the implant [34].

New bone formation in the oriented scaffolds based on the available pore area (volume) was 55 ± 5% in comparison with 46 ± 13% for the trabecular scaffolds. Less new bone formed in the trabecular scaffolds, thus suggesting again that the pore channel tortuosity played a crucial role in adjusting bone infiltration in the scaffolds [34].

In addition, Bi et al. [36] studied the effect of the microstructure of the scaffolds on bone regeneration by using a rat calvarial model [36]. Scaffolds of 13-93B3 glass and Cu-doped 13-93B3 glass fabricated with three different microstructures, described as trabecular, oriented, and fibrous, were implanted in rat calvarial defect models and compared [36].

The trabecular scaffolds of 13-93B3 glass exhibited a higher quantity of new bone growth and greater osteoinductive ability in comparison to the oriented or fibrous scaffolds of the same glass [36]. This difference in bone formation may be attributed to the higher porosity (77%) and larger pore size (200–400 µm) of trabecular scaffolds in comparison to the other scaffolds. Indeed, large and interconnected pores in the scaffold may have led to increased bone ingrowth, since their presence may have allowed greater migration and proliferation of mesenchymal cells, osteoblasts, and neovascularization, resulting in a higher oxygen tension and nutrient supply. The lower porosity, as well as pores of size <100 µm may have impeded tissue penetration into the oriented or fibrous scaffolds [59].

The new bone either penetrated from the edge of the defect, grew along the dural side of the defect, or was formed by a combination of both [59]. Unlike the previous study by Liu et al. [34], in this study bone growth in the trabecular scaffold was not limited to the periphery of the implant [36]. This is attributable to the different tortuosity of the scaffold pore channels.

Numerous literature studies have shown that Cu ions accelerate angiogenesis [118,119], and angiogenesis and bone regeneration ability are interrelated. Moreover, it has been found that Cu^+^ and Cu^2+^ are able to kill bacteria through the generation of ROS, lipid peroxidation, protein oxidation, and DNA degradation [120]. 

Enhanced angiogenesis leads to a better bone regeneration ability because it is believed that the newly formed vessels provide a rapid blood supply, offering the cell more nutrition for new bone formation [39]. However, in the present investigation, the Cu-doped 13-93B3 scaffolds did not show a significant increase in total new bone growth compared with 13-93B3 scaffolds. A considerable difference was found only between the Cu-doped and undoped scaffolds with fibrous microstructure [36].

A significant achievement is that, with the exception of 13-93B3 fibrous scaffolds, the amount of new bone formed in the rat calvarial model implanted with 13-93B3 and Cu-doped 13-93B3 scaffolds was larger than in the defects implanted with particles of 45S5 Bioglass^®^ that were used as positive control [36]. This is in accordance with the results of the previous study of Bi et al. [121], which showed that bioactive borate glass scaffolds had a higher bone regeneration ability than bioactive silicate glass scaffolds [121].

**Figure 18:**
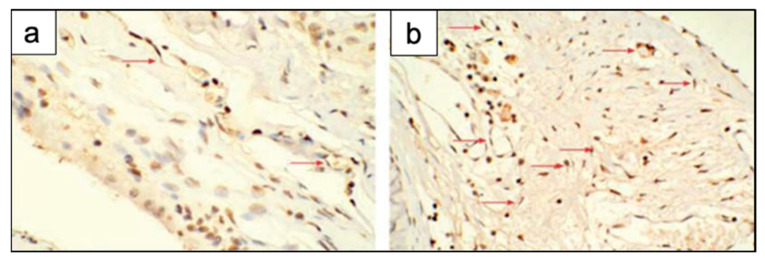
Immunohistochemical staining in rat calvarial defects implanted with (**a**) BG scaffolds and (**b**) Cu-doped BG scaffolds at 8 weeks post-implantation. There were more new blood vessels (red arrows) in the defects implanted with the Cu-doped BG scaffolds. Figure reproduced from Ref. [39] with permission from the Royal Society of Chemistry.

Not only was 13-93B3 borate glass tested in vivo in a rat calvarial defect model, but also D-Alk-B borate glass [39,41]. D-Alk-B borate glass was doped with copper in order to enhance angiogenesis [39]. Contrary to the previous study of Bi et al. [36], in which no differences were found in bone growth between Cu-containing and Cu-free scaffolds, in the present study Cu-doped D-Alk-B scaffolds exhibited a greater ability to stimulate angiogenesis and regenerate bone in comparison to the undoped glass scaffolds (Figure 18) [39].

The same research group also tested D-Alk-B scaffolds loaded with Fe3O4 [40] and ZnO [41] in vivo in rat calvarial defect models. 

**Figure 19:**
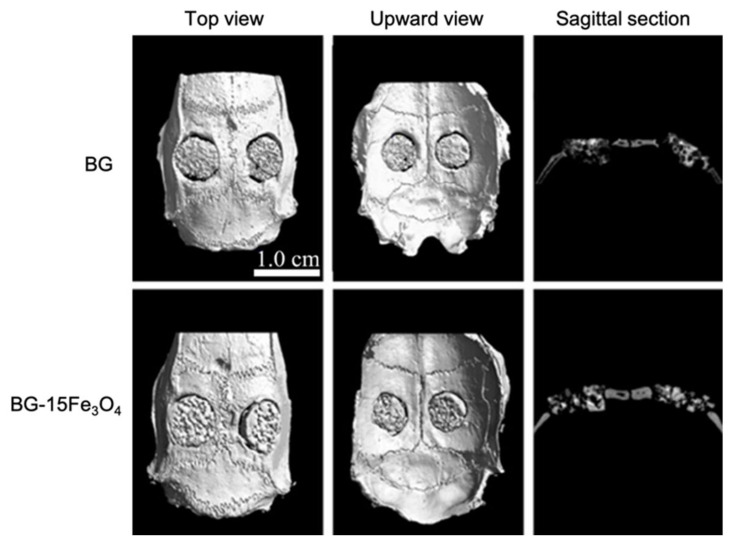
Micro-CT evaluation of bone regeneration in the rat calvarial defects implanted with borosilicate BG scaffolds and borosilicate BG scaffolds loaded with Fe_3_O_4_ magnetic nanoparticles (MNPs). Figure reproduced from Ref. [40] with permission from the Royal Society of Chemistry.

The outcomes showed a higher quantity of new bone in the defects implanted with scaffolds loaded with Fe_3_O_4_ magnetic nanoparticles (MNPs) than ones implanted with scaffolds alone (Figure 19) [40].

Indeed, the presence of Fe_3_O_4_ MNPs probably resulted in many miniature magnetic forces in the scaffold under the external magnetic field, thus steadily spurring osteoblastic cell proliferation, differentiation, osteogenic gene expression, and secretion of new natural extracellular matrices. In addition, free iron released into the cytoplasm could have led to a better cell cycle progression in vivo [40].

**Figure 20:**
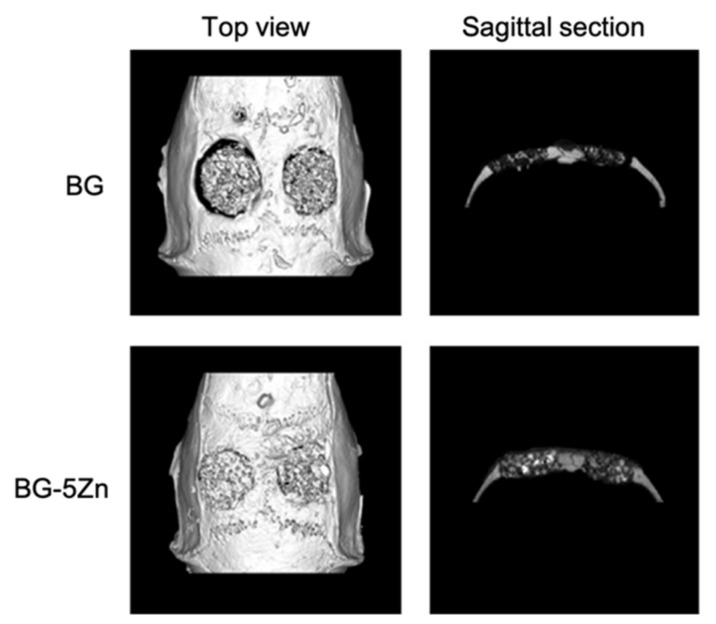
Micro-CT evaluation of bone regeneration in the rat calvarial defects implanted with the borosilicate BG scaffolds and Zn-doped borosilicate BG scaffolds at 8 weeks post-implantation. Figure reproduced with permission from Wang et al. [41].

Zn-doped D-Alk-B scaffolds showed an improved bone regeneration compared to the non-doped scaffolds (Figure 20), too. This finding proved that Zn-doped scaffolds have high osteogenic ability, making them promising materials for bone tissue repair and regeneration [41]. Moreover, it was reported that the release of Zn^2+^ ions from the glass produces an antibacterial effect via the production of ROS and the accumulation of these species in the cytoplasm or on the outer membranes of bacteria [120].

**Figure 21:**
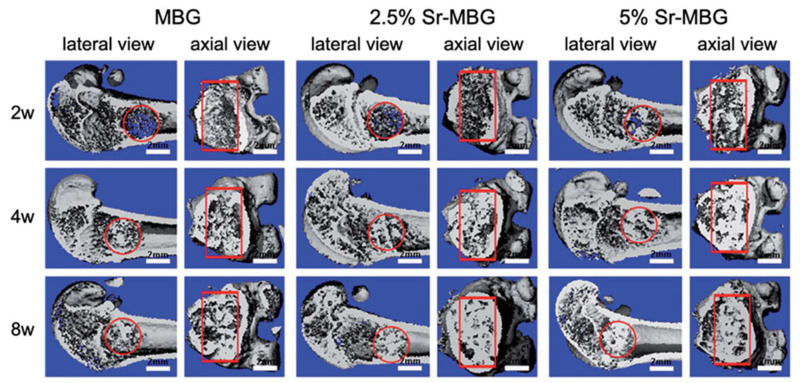
3D reconstruction of longitudinal section and cross-section images by micro-CT at 2-, 4- and 8-weeks post-implantation of MBG scaffolds and MBG scaffolds containing strontium in the critical femoral defect. The red circle and rectangle indicate the boundary of the defects. Figure adapted from Zhang et al. [43] with permission from the Royal Society of Chemistry.

The rat was also chosen for in vivo testing of mesoporous bioactive glass scaffolds [43,44,45]. Zhang et al. [43] incorporated strontium into mesoporous bioactive glass (Sr–MBG) scaffolds in order to combine the therapeutic action of Sr^2+^ ions on osteoporosis with the bioactivity of MBG to regenerate osteoporotic-related fractures [43]. MBG scaffolds and Sr–MBG scaffolds were implanted in critical-sized femur defects of ovariectomized rats to study in vivo regeneration of osteoporotic bone defects [43]. Sr–MBG scaffolds showed greater new bone formation than MBG scaffolds alone. Moreover, incorporation of Sr into MBG scaffolds led to much more mineralization and density (Figure 21) [43]. Indeed, it was found that Sr–MBG scaffolds spurred a more effective osteoconductive and anti-osteoporotic phenotype, which increased the speed and quality of bone regeneration and remodeling and changed the trabecular morphology from a more rod-like aspect to the plate-like appearance with retrieval of trabecular connectivity [43].

The results also showed that Sr release in blood was maintained at a very low level, which might suggest the slightest chance of side effects on overall health. In addition, the Sr, Si, and Ca ions in urine were higher than those in blood, meaning that the ionic products from Sr–MBG scaffolds can be metabolized and excreted by urine [43]. Therefore, these findings suggested that Sr–MBG scaffolds are suitable biomaterials for regenerating osteoporosis-related fractures by the release of Sr-containing ionic products [43].

In another study, the same research group investigated if the Sr-containing MBG scaffolds were also advantageous for the repair of alveolar bone defects created in periodontal tissues of ovariectomized rats [36].

The outcomes showed that Sr–MBG increased alveolar bone regeneration in periodontal tissues in ovariectomized rats when compared to MBG alone [36]. For the first time, the capacity of scaffolds containing strontium to promote bone formation in periodontal tissue in vivo was demonstrated [36]. The release of Sr^2+^ ions enhanced osteoblast function by augmenting new bone formation. In addition, it was also found that the number of multi-nucleated osteoclasts was substantially reduced when Sr was added in MBG scaffolds [36]. This finding agrees with literature studies that proved that Sr is able to suppress osteoclastogenesis [122]. Taken together, these findings proposed that the implant of Sr-containing scaffolds may lead to a better healing of periodontal defects caused by osteoporosis [36].

Besides Sr–MBG [43], the 45Ca–MBG scaffolds produced by Sui et al. [45] were also implanted in critical-sized rat femur defects [45]. The outcomes showed that after scaffold implantation the mesoporous structure sped up the local ion exchange. Reactive ions such as Si and Ca released from MBG fostered bone regeneration in vivo. However, it was found that only a small quantity of MBG-released calcium ions was transformed into calcium components of the new bone matrix [45]. The rat model has several limits, such as the small size that makes it unsuitable for testing multiple implants [112]. In addition, the long bones of rats are small and characterized by slim and weak cortices and do not exhibit haversian-type cortex remodeling, as in bigger animals [25].

In a very recent study, cobalt-doped scaffolds fabricated with the foam replica method were tested in vivo using rat calvarial defect models [123]. Although commonly considered a toxic element [124], it was demonstrated that the release of Co ions below the toxicity limit can induce a hypoxia-like response, stimulating the production of certain kinds of angiogenic factors. More specifically, the study investigated the mechanism of cobalt-doped bioactive borosilicate (36B_2_O_3_, 22CaO, 18SiO_2_, 8MgO, 8K_2_O, 6Na_2_O, 2P_2_O_5_; mol %) glass scaffolds for bone tissue repair and blood vessel formation in the critical-sized cranial defect site of rats, as well as their effects on human bone marrow-derived mesenchymal stem cells (hBMSCs) in vitro [123]. The obtained scaffolds efficiently controlled the release of Co^2+^ ions from their surface without producing any cytotoxic effect. Moreover, it was demonstrated that cobalt release improves HIF-1α generation, vascular endothelial growth factor (VEGF) protein secretion, and alkaline phosphatase (ALP) activity and upregulates the expression of osteoblast and angiogenic relative genes. Eight weeks after implantation, the bioactive glass scaffolds with 3 wt % CoO remarkably enhanced bone regeneration and blood vascularized networks at the defective site, showing great potential as bone graft material mimicking hypoxia conditions [123]. 

**Figure 22:**
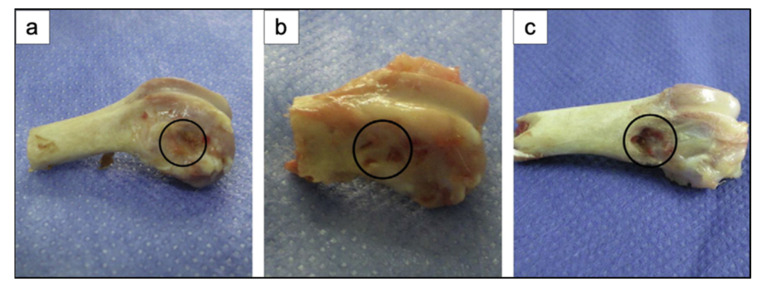
Gross appearance of rabbit femoral head defects implanted with 13-93B1 scaffolds for (**a**) 4 weeks, and (**b**) 8 weeks; (**c**) unfilled defect at 8 weeks. Figure reproduced with permission from Gu et al. [37] (Copyright © 2021 Elsevier B.V.).

An alternative to the rat model is the rabbit model. In particular, New Zealand white rabbit is considered the second animal model of choice to test in vivo biomaterials for musculoskeletal investigations [114]. Indeed, it is easily available, it has a small size which makes it easy to handle and house. Moreover, similarities in bone mineral density and fracture toughness of mid-diaphyseal bone between humans and rabbits were found [125]. For instance, Gu et al. [37] evaluated the ability of 13-93B1 scaffolds to promote bone regeneration in vivo using a non-critical-sized defect created in the femoral head of rabbits [37]. Figure 22 exhibits the defects implanted with the 13-93B1 scaffolds after 4 and 8 weeks and the unfilled defects at 8 weeks. The defects implanted with the scaffolds presented better bone healing than the unfilled defects. Moreover, as the scaffold’s implantation time increased, bone healing increased. These results suggest that the trabecular structure of 13-93B1 scaffolds produced in the present study is favorable for bone ingrowth [37]. In particular, the results showed that at 8 weeks post-implantation the scaffolds were partly converted to HA and they were well integrated with newly formed bone [37]. It is well known that 13-93B1 glass converts to HA faster than the silicate 13-93 glass and slower than the borate 13-93B3 glass [35]. Accordingly, scaffolds of 13-93B1 might offer an excellent combination of conversion to hydroxyapatite and retention of the strength, which is difficult to achieve with 13-93 or 13-93B3 glass [37].

**Figure 23:**
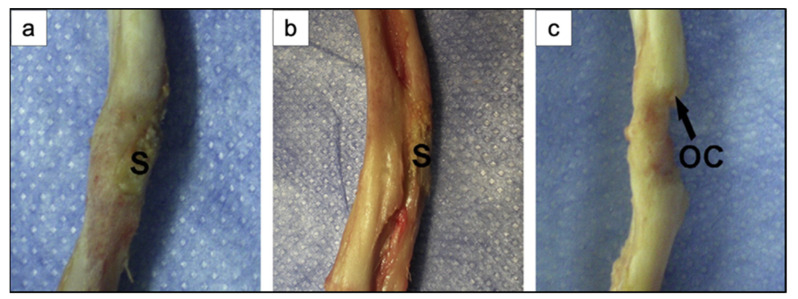
Gross appearance of segmental defect sites in rabbit radii after implantation for 8 weeks with (**a**) 13-93B1 scaffolds, and (**b**) 13-93B1 scaffolds loaded with PRP; (**c**) unfilled defect at 8 weeks. S: scaffold; OC: osseous callus. Figure reproduced with permission from Gu et al. [37] (Copyright © 2021 Elsevier B.V.).

Then 13-93B1 scaffolds were loaded with platelet-rich plasma (PRP), and both the loaded scaffolds and the unloaded ones were implanted in segmental defects in the diaphysis of rabbit radii to evaluate their ability to support bone regeneration in vivo [37]. Figure 23 displays the segmental defects in the rabbit radii implanted with 13-93B1 scaffolds, 13-93B1 scaffolds loaded with PRP, and the unfilled defects at 8 weeks [37]. Both kinds of scaffolds integrated with the host bone, and the formation of callus seemed to start from both defect ends (Figure 23a,b). On the contrary, no new bone was present in the unfilled defect (Figure 23c) [37].

These findings suggested that both kinds of scaffolds supported new bone formation in rabbit defects. However, the scaffolds loaded with PRP exhibited an increased ability to support the healing and the bone formation in comparison with the unloaded ones [37].

Indeed, PRP contains several growth factors, such as transforming growth factor β1, vascular endothelial growth factor, and insulin-like growth factor, which are known to stimulate the proliferation and differentiation of osteoblastic cells. Therefore, it is believed that the growth factors impart the osteoinductive property to PRP-loaded scaffolds, besides the inherent osteoconductive property of the porous scaffolds themselves [37]. The 13-93B1 scaffold used in the present study was proved to be an effective vector for PRP, which, being characterized by limited stability and a short life span, has to be released in a controlled manner. 

Like PRP, the recombinant human bone morphogenetic protein-2 (rhBMP-2) is also known to increase the bone formation capacity [126]. This was also proved by Tang et al. [51], who developed trimodal macro/micro/nanoporous scaffolds loaded with rhBMP-2 (TMS/rhBMP-2) [51]. TMS/rhBMP-2 was implanted in thigh muscle pouches of mice and in rabbit radius critical-sized defects to evaluate in vivo the osteogenic performance of this structure [51]. The outcomes showed that TMS/rhBMP-2 had a great bone regeneration ability. In particular, this scaffold resulted in the extensive regeneration of critical-sized defects with rapid medullary cavity reunion and sclerotin maturity [51].

In accordance with the theories of endochondral bone formation [127,128], the regeneration process in this work can be divided into three phases: inflammation and cartilage formation (0–2 weeks), primary bone formation (2–4 weeks), and bone remodeling (after 4 weeks). Since over 90% of the scaffold degraded by week 8, it was assumed that the osteogenic promotion of TMS/rhBMP-2 took place especially in the first 8 weeks after implantation. On the basis of these outcomes, TMS/rhBMP-2 is considered a promising bone substitute for clinical applications [51].

The rabbit model suffers from limitations including the high bone turnover rate and the fast skeletal transformations, which make it difficult to apply the achievements obtained from rabbit investigations to humans [25]. Larger animals are more like humans. For example, pigs are the animals with bone regeneration processes most similar to human ones. However, no reports on the in vivo study of the bone regenerative ability of bioactive glass scaffolds in pigs was found. This may be due to the high body weight and growth rate of the pig and its difficult management [114,115].

Dogs are the most frequently used animals for musculoskeletal and dental investigation [129]. However, dogs’ bones have higher bone mineral density, fracture toughness, and different microstructure compared to human ones [125]. In addition, the use of dogs for in vivo studies also involves ethical problems since the dog is considered a pet [130].

However, Lee et al. [30] implanted calcium phosphate glass scaffolds in 1-wall intra-bony defects of beagle dogs to evaluate the in vivo bone regeneration ability [30]. The defects implanted with calcium phosphate glass scaffolds and chitosan membranes showed a much larger amount of new cementum and alveolar bone regeneration compared to empty controls treated with the chitosan membrane alone. These findings suggested that calcium phosphate glass is a promising material for new bone and cementum regeneration [30].

## 6. Conclusions

Since 2006, the foam replica method has been used extensively to fabricate scaffolds for bone tissue engineering application. The high versatility of the method, together with the ease of execution and the cost effectiveness, make this technique one of the most appealing manufacturing processes for producing porous and trabecular-like 3D structures perfectly resembling natural bone.

As reported by numerous literature studies, scaffolds produced with the foam replica method exhibit not only an exceptional morphological similarity with spongy bone, but also mechanical strength and mass transport properties matching those of human bone tissue, despite the considerable variability within wide ranges, which actually constitutes a significant obstacle to their clinical validation. 

Nevertheless, numerous in vivo studies performed on several animal models strongly support the exceptional potential of these scaffolds, thus motivating additional efforts toward the standardization of the process in order to improve the reproducibility and the reliability of foam-replicated artificial bone substitutes to enable their application in the management of bone defects, thus closing the gap between laboratory experiments and clinical treatment. 

## Figures and Tables

**Table 1:** Summary of natural marine sponge architecture properties. Table adapted from Boccardi et al. [12].

Property	SL	SA
Pore dimension inhalant surface	1.0 ± 0.1 mm	590 ± 50 µm
Pore dimension exhalant surface	1.7 ± 0.2 mm	920 ± 90 µm
Pore wall thickness	540 ± 80 µm	400 ± 40 µm

**Table 2:** Pore characteristics and compressive strength of several bioactive glass scaffolds produced with the foam replica method.

Glass System	Template	Pore Size (µm)	Porosity (%)	Compressive Strength (MPa)	Reference
45S5 Bioglass^®^: 45SiO_2_–24.5CaO–24.5Na_2_O–6P_2_O_5_ wt %	Polyurethane	510–720	~90	0.3–0.4	Chen et al. [2]
45S5 Bioglass^®^: 45SiO_2_–24.5CaO–24.5Na_2_O–6P_2_O_5_ wt %	Polyurethane	100–600	~70	~2.5	Baino et al. [27]
45S5 Bioglass^®^: 45SiO_2_–24.5CaO–24.5Na_2_O–6P_2_O_5_ wt %	*Spongia Aagaricina* (SA sponge)	1–600	68.0 ± 0.2	1.8 ± 0.3	Boccardi et al. [12]
45S5 Bioglass^®^:45SiO_2_–24.5CaO–24.5Na_2_O–6P_2_O_5_ wt %	*Spongia Lamella* (SL sponge)	1–900	76 ± 2	4.0 ± 0.4	Boccardi et al. [12]
47.5. B: 47.5SiO_2_–20CaO–10MgO–2.5P_2_O_5_–10K_2_O–10Na_2_O mol %	Stale bread	-	72.0 ± 1.5	0.62 ± 0.20	Fiume et al. [10]
CEL2: 45SiO_2_–3P_2_O_5_–26CaO–7MgO–15Na_2_O–4K_2_O mol %	Polyurethane	100–600	66.4 ± 2.0	4.5 ± 0.9	Baino et al. [27]
CEL2: 45SiO_2_–3P_2_O_5_–26CaO–7MgO–15Na_2_O–4K_2_O mol %	Polyurethane	100–500	~70	1.0 ± 0.4	Vitale-Brovarone et al. [29]
I-CEL2: 3SiO_2_–45P_2_O_5_–26CaO–7MgO–15Na_2_O–4K_2_O mol %	Polyurethane	100–500	82.0 ± 6.7	0.4 ± 0.2	Baino et al. [9]
CaO–CaF_2_–P_2_O_5_–MgO–ZnO	Polyurethane ester	500–800	-	~1.5	Legeros et al. [3]
SCNA: 57SiO_2_–34CaO–6Na_2_O–3Al_2_O_3_ mol %	Polyurethane	~240	56 ± 6	18 ± 5	Baino and Vitale-Brovarone [31]
13-93: 53SiO_2_–20CaO–6Na_2_O–12K_2_O–5MgO–4P_2_O_5_ wt %	Polyurethane	100–500	85 ± 2	11 ± 1	Fu et al. [33]
13-93B3: 6Na_2_O–7.9K_2_O–7.7MgO–22.1CaO–54.6B_2_O_3_–1.7P_2_O_5_ mol %	Polyurethane	100–500	82 ± 3	5.0 ± 0.5	Fu et al. [35]
D-Alk-B: 6Na_2_O–8K_2_O–8MgO–22CaO–18SiO_2_–2P_2_O_5_–36B_2_O_3_ mol %	Polyurethane	100–500	67.7 ± 2.6	9.7 ± 1.3	Liu et al. [30]
45S5 Bioglass^®^: 45SiO_2_–24.5CaO–24.5Na_2_O–6P_2_O_5_ wt %	Polyurethane	~470	85 ± 2	~1.5	Bretcanu et al. [6]
45S5 Bioglass^®:^ 45SiO_2_–24.5CaO–24.5Na_2_O–6P_2_O_5_ wt %	Polyurethane	400–700	79 ± 1	0.24 ± 0.06	Balasubramanian et al. [47]
65SiO_2_–15CaO–18.4Na_2_O–0.1MgO–1.5B_2_O_3_ wt %	Polyurethane	110–550	82 ± 2	1.0 ± 0.3	Erol et al. [48]
58S: 58SiO_2_–33CaO–9P_2_O_5_ wt %	Demineralized Bone Matrix	300–700	89.3 ± 2.0	0.16 ± 0.05	Xia et al. [64]
58S-Gelatin: 58SiO_2_–33CaO–9P_2_O_5_ wt %, gelatin > 10 wt %	Demineralized Bone Matrix	300–700	87.7 ± 1.1	4.9 ± 0.2	Xia et al. [64]
5.0Silk–MBG: SiO2–CaO–P2O5	Polyurethane	200–400	~94	~0.25	Wu et al. [42]
